# Ultra-processed food intake and its nutritional consequences among youth when consuming vegan, lacto-ovo-vegetarian, pescatarian or omnivore diets: a cross-sectional study

**DOI:** 10.1186/s12937-026-01378-8

**Published:** 2026-08-04

**Authors:** Anna Turesson Wadell, Isabelle Mulkerrins, Christel Larsson

**Affiliations:** Department of Food and Nutrition, and Sport Science, University of Gothenburg, PO Box 300, Gothenburg, SE-405 30 Sweden

**Keywords:** Plant-based diet, Ultra-processed foods, Nutrition, Dietary assessment, Youth

## Abstract

**Background:**

Dietary recommendations promote a predominantly plant-based diet, and the growing interest in such diets has resulted in an increased supply of ultra-processed plant-based alternatives to animal-based foods such as milk and meat. However, most evidence points towards adverse health effects of a high intake of ultra-processed foods (UPF). The purpose of this study was to investigate differences in UPF intake and its nutritional consequences in a youth population in Sweden consuming vegan, lacto-ovo-vegetarian, pescatarian, or omnivore diets.

**Methods:**

In total, 244 participants, 16–24 y old, participated in the cross-sectional study VeggiSkills-Sweden. Foods reported in four 24-h recalls were classified into UPF or non-UPF according to the Nova classification system. Percentage contribution of UPF to energy, nutrients, and other food components was investigated and compared between the four dietary groups. Associations between UPF intake and demographic variables, health-related lifestyle factors and nutrient status were analysed.

**Results:**

Vegans obtained more energy, nutrients (e.g., n-3 fatty acids, vitamin D, B_12_, calcium and zinc), and salt from UPFs compared to the other dietary groups but did not have a higher energy intake or higher body mass index. They consumed more total dietary fiber and polyunsaturated fatty acids (PUFA), and less saturated fatty acids (SFA). Also, vegans obtained all vitamin D and B_12_ from UPFs. Across the entire study population, participants with the highest intake of UPF had a lower level of general nutrition knowledge and consumed less whole grain and more free sugars, but less SFA and more PUFA. UPF intake was not associated with biochemical indicators of nutritional status.

**Conclusions:**

Youths adhering to a vegan diet obtained more energy and nutrients, including nutrients of concern in a plant-based diet, from UPFs than youth adhering to lacto-ovo-vegetarian, pescatarian or omnivore diets. As UPFs are defined today, authorities should be careful discouraging UPF intake while at the same time promoting a more plant-based diet among youths.

**Supplementary Information:**

The online version contains supplementary material available at 10.1186/s12937-026-01378-8.

## Introduction

Adverse effects of climate change and increased awareness of the positive health effects of plant-based foods have prompted a growing interest in plant-based diets. According to the Nordic nutrition recommendations (NNR) 2023, it is recommended to consume a predominantly plant-based diet that is rich in fruit and vegetables, legumes, whole grain, nuts and fish, and with a limited amount of red meat and poultry [1]. Such a diet promotes health through lowering the risk of non-communicable diseases such as cardiovascular disease, type 2 diabetes and certain types of cancer while also reducing environmental footprint [1]. Vegetarian diets, where meat, poultry, fish, and sometimes also dairy and egg, are completely omitted, are also known for their health-promoting effects and low climate impact [2]. However, there is a risk of adverse nutritional consequences when omitting entire animal-based food groups from the diet if not replaced with equally nutritious plant-based foods. Vitamin B_12_, vitamin D, calcium, iron, zinc and long-chained n-3 fatty acids are some examples of nutrients of concern in a vegetarian diet [2]. Nonetheless, the plant-based food market has expanded considerably in recent years [3]. There are now a variety of plant-based food items such as dairy substitutes and plant-based meat analogues (PBMAs), of which some are fortified with micronutrients to mimic the nutrient content of the animal-based alternatives. Such products are most often classified as ultra-processed foods (UPFs) [4–6].

Monteiro and co-authors [7] define UPFs as ‘…formulations of ingredients, mostly of exclusive industrial use, that result from a series of industrial processes…’. Most foods classified as UPFs, such as candies, soft drinks, packaged salty snacks, mass produced bread, breakfast cereals and ready-to-eat meals, are energy-dense and rich in fat, sugar, and salt, and contain additives, e.g., emulsifiers, colors and flavors, or ingredients of no culinary use, e.g., hydrolyzed proteins and modified oils [7]. Several observational studies have shown associations between UPF intake and risk of obesity, type 2 diabetes, cardiovascular disease and mortality [8]. The few randomized controlled trials performed have demonstrated that consumption of UPF leads to higher energy intake, higher eating rate and weight gain compared to consumption of minimally processed foods in adults [9, 10]. On the other hand, there are also industrially manufactured food items that are nutrient dense, contain less energy, and traditionally are considered healthy, but because they contain additives or ingredients of no culinary use are classified as UPFs. Whole grain bread and breakfast cereals, fortified dairy substitutes, and protein rich (sometimes fortified) PBMAs, are examples of UPFs that contribute with important nutrients, particularly in plant-based diets [2, 11].

In the early 2000’s, a Swedish study investigated food choices and nutrient intake of young people consuming plant-based diets [12, 13]. Since then, further recommendations to reduce meat intake and increase plant-based food intake have been announced and consequently the plant-based food market, including plant-based UPFs, has evolved. There is a need to investigate the nutritional consequences of the food choices young people of today make when consuming different plant-based diets.

### Aim

To investigate differences in UPF intake and its nutritional consequences in a youth population in Sweden consuming vegan, lacto-ovo-vegetarian, pescatarian, or omnivore diets.

## Materials and methods

### Study design and population

The cross-sectional study VeggiSkills-Sweden was carried out in Gothenburg, Sweden between 2022 and 2024. It aimed to assess the nutritional consequences of consuming different plant-based diets in a youth population, and to explore their food literacy, and attitudes in relation to their dietary practice. A total of 244 individuals were recruited to the study using convenience and snowball sampling through various recruitment methods (e.g., posters in universities, schools, training facilities, social media [Facebook and Instagram], newsletters, and through student-organized sports and sustainability events). All participants visited a research facility at the University of Gothenburg once to complete several measurements on site. The study design and data collection methods have been thoroughly described elsewhere [14].

In a web-based questionnaire, the participants self-defined their current dietary regimen (vegan, ovo-vegetarian, lacto-vegetarian, lacto-ovo-vegetarian, pescatarian, flexitarian, or omnivore diet) and answered questions regarding consumption of dairy, egg, fish, poultry, and red meat for the last 6 months. Based on their reported consumption, the participants were categorized into consuming a vegan diet (no animal products), a lacto-ovo-vegetarian diet (including dairy and/or egg), a pescatarian diet (including fish/seafood and possibly dairy and/or egg in the diet, but no meat) or an omnivore diet (including meat and other animal-sourced foods in the diet). More details regarding the categorization have been published previously [14]. Exclusion criteria were chronic or acute disease, and pregnancy or lactation. To be included in the VeggiSkills-Sweden study, participants had to be 16–24 years old, have adhered to the diet (vegan, lacto-ovo-vegetarian, pescatarian, or omnivore) for ≥ 6 months, comprehend Swedish, and be able to visit the research facility to complete measurements on site.

### Background characteristics

Information about dietary habits (e.g., adherence to dietary regimen, habitual supplemental use), health-and lifestyle habits (e.g., physical activity level) and food literacy competencies (e.g., general nutrition knowledge), and parental education was collected through a 255-item web-based questionnaire.

The following question about adherence was asked: “For how long have you followed your current dietary regimen?” with the response alternatives “Less than 6 months”, “6–11 months”, “1–3 years”, “3–5 years”, “>5–9 years”, “10 + years”, and “My whole life”. These response alternatives were combined into four categories (< 1 year, 1–5 years, > 5–9 years and ≥ 10 years) for description of the study population, and two categories (< 5 years and ≥ 5 years) for the statistical tests when needed.

Regarding supplemental use, participants were asked to report their intake frequency of several vitamins and minerals, omega-3, kelp and nutritional yeast in the past 6 months. If the participant used another supplement than those inquired about, it could be specified in an open-ended free-text question. They were instructed to report all supplements used. In the present paper, reported intake was converted to supplement user yes/no meaning that type of supplement or frequency were not reported.

The question on parental education was divided into mother’s and father’s education, with response alternatives “Less than 12 years of school (no upper secondary diploma)”, “12 years of school (upper secondary diploma)”, “University college/University less than 3 years”, “University college/University3 years or more”, and “Other/Non applicable”. In this paper, only the proportion of “University college/University 3 years or more” were reported.

In the questionnaire, participants reported their frequency of moderate intensity and vigorous intensity exercise per week in the last six months. This was converted to an individual physical activity level (PAL): PAL 1.4 = < 1 time/week of moderate intensity, PAL 1.55 = 1–2 times/week of moderate intensity, PAL 1.7 = 3–4 times/week of moderate intensity, PAL 1.8 = 5–6 times/week of moderate intensity, PAL 2.0 = ≥ 6 times/week of moderate intensity + 1–2 times/week of vigorous intensity.

### General nutrition knowledge

An adapted version of the validated “General nutrition knowledge questionnaire revised” (GNKQ-R) [15] was used to measure the participants’ general nutrition knowledge. The questionnaire was translated into Swedish, shortened, and some questions were adapted to better suit the VeggiSkills-Sweden study (manuscript under review). The adapted questionnaire contained 29 questions (including multiple item questions) regarding dietary recommendations, food groups and food choices. Correct answers yielded 1 point while answering incorrectly or “unsure” yielded 0 points. Possible scores ranged from 0 to 72, where a higher score indicated higher level of knowledge. A further categorization into low, moderate and high level of general nutrition knowledge was made for descriptive purposes using Blooms cut-offs [16]. A low level corresponded to 0–42 points, a moderate level to 43–57 points, and a high level to 58–72 points [17].

### Anthropometrics

Weight was measured to the closest 0.1 kg (light clothing and no shoes) using a Beurer 180BF digital scale (Beurer GmbH, Germany). Height was measured to the closest 0.1 cm using a wall-mounted stadiometer (Hyssna M, Sweden). Body mass index (BMI) was calculated as kg/m^2^ using the World Health Organization’s (WHO) classification for underweight (< 18.5), normal weight (18.5–24.9) and pre-obesity/obesity (≥ 25) [18].

### Dietary assessment

Dietary intake data was collected from two up to four 24-h recalls performed on non-consecutive days and each participant completed their 24-h recalls within 2–7 weeks. Of these, 1–2 days were weekend days. The first 24-h recall was performed as an interview during the study visit, following the Multiple Pass method [19]. The interviewer registered the dietary intake in the nutrition analysis program Nutrition Data (Nutrition Data Sweden AB, Sweden). For the remaining three recalls, the participants were asked to report their intake themselves using the same program. They received a text message and e-mail with instructions asking them to report yesterday’s intake. The days on which they were to do this were unannounced. Nutrition Data is connected to the Swedish Food Composition Database including ~ 2300 generic food items (version 2023 06 13, Swedish National Food Agency) which the participants were instructed to preferably report foods from. However, they were also allowed to use Nutrition Data’s own food composition database. This database, including ~ 3400 different foods (both generic food items and specific brands) and recipes, was a complement to the Swedish Food Composition Database. The foods in this database have either a calculated nutrient value or are, in a few cases, retrieved from the Finnish, Norwegian or American Food Composition Database. The database is continuously updated and therefore has no version number.

The nutrient values in the Swedish Food Composition Database are either based on analyses performed by the Swedish National Food Agency (most values), values borrowed from e.g., the food industry, publications or other countries’ food tables, estimated values or calculated values [20]. Examples of estimated values are values for whole grain and free sugars. The amount of whole grain and free sugars is estimated from e.g., ingredient lists since it cannot be analysed. Whole grain is calculated as the sum of the estimated values for whole grain (defined as grains containing all parts of the kernel) in oats, wheat, rye, barley and other grains but not in pseudograins [21]. Free sugars are defined as added sugars and naturally occurring sugars in honey, syrup, fruit juices and fruit juice concentrate and the method for estimating these values has been described by Wanselius et al. [22].

Participants were instructed to report intake as detailed as possible, e.g., ingredients used. There was an open textbox in Nutrition Data where participants could leave notes about their intake. When estimating portion sizes, participants were instructed to use the booklet *Portionsguide* (“Portion guide”) published by the Swedish National Food Agency. Since some commonly eaten snack foods were missing in this booklet, portion size photos of seven other food items (e.g., popcorn, ice cream, candy) were added [14, 23]. Participants could report portion sizes in household measures, grams, or slice/piece/etc. More details about the interview and the reporting of dietary intake have been published previously [14].

When reviewing the reported intake, study staff corrected obvious input mistakes, e.g., 10 tbsp of sandwich spread was changed to 10 g. When participants were unsure of how to register a certain food, they had the possibility to leave a note in the open textbox with details of the food so the study staff could choose the most similar food from the Swedish Food Composition Database.

Participants reported their intake of dietary supplements in the 24-h recalls, but since the present paper focused on UPF intake, these were excluded from the analyses. However, certain food items that could be considered dietary supplements, i.e., protein powder, protein enriched food items, nutritional yeast (unless specified as capsules), micronutrient fortified juice, and energy drinks, were included. In a few cases, nutrient supplements were incorrectly left in the dataset. If it affected the result greatly, the participant was removed from the analysis of that specific nutrient. To describe the characteristics of the study population, habitual supplement use reported in the web-based questionnaire was used.

In a previous publication from VeggiSkills-Sweden investigating usual dietary intake [14], two participants were excluded from analyses due to performing only one 24-h-recall since this is not enough for calculation of usual intakes. To maintain consistency, these two participants were excluded also from the analyses in the present paper.

### Classification of ultra-processed foods (UPFs)

**Fig. 1:**
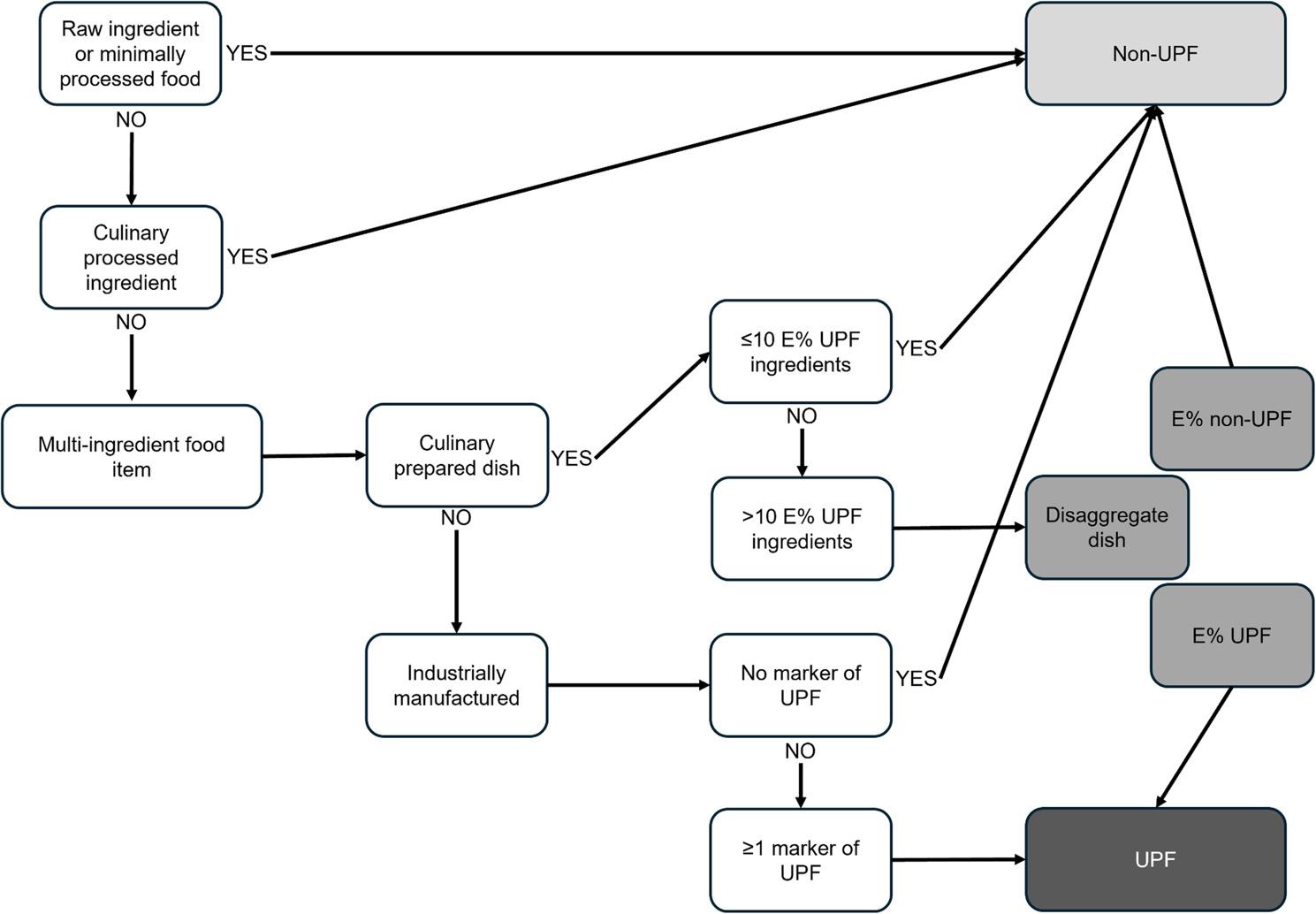
Flow chart of the UPF classification procedure of reported dietary intake in the VeggiSkills-Sweden study. The method used to identify and classify UPF was inspired by an unpublished analysis plan by Sällström and Bärebring. UPF Ultra-processed food

All reported food items were classified into UPF or non-UPF using the Nova Classification system [7]. This was performed by one dietitian (the first author; ATW). In the Nova Classification system, foods are classified into one of four possible groups; Group 1: Unprocessed or Minimally processed foods (processed by, e.g., removing inedible parts, boiling or freezing, often to extend storage life), Group 2: Processed culinary ingredients (e.g., salt, oil, sugar, butter), Group 3: Processed foods (industrial manufactured products where foods from group 2 have been added to foods in group 1 for preservation or fermentation), Group 4: Ultra-processed foods (“[…] formulations of ingredients, mostly of exclusive industrial use, that result from a series of industrial processes […] [7], such as ready-to-eat meals, soft drinks, candies, margarine, nuggets, breakfast cereals, certain pre-packaged bread, PBMAs, etc.). Figure 1 shows an overall description of the classification steps, and for the present study, non-UPF items were foods belonging to groups 1–3. The method used to identify and classify UPF in the present study was based on previous publications regarding practical application of the Nova classification system [7, 24, 25]. A general assumption during the classification was that food items industrially manufactured and with ≥ 1 UPF-marker in the ingredient list, were considered ultra-processed [7]. UPF markers are substances rarely used in the kitchen at home (e.g., vegetable protein isolates, maltodextrin etc.) or additives which make the food more appealing (colors, emulsifiers, flavor enhancers etc.).

#### Conservative classification of UPFs for main analyses

For the main analyses, a conservative approach in UPF classification was undertaken. This means that all composite dishes and bakery goods which were not further specified in product name or the participant’s own note, were identified as culinary preparations, i.e. made from scratch at home or at a restaurant (not a fast-food chain outlet) or bakery. These items/dishes were classified as non-UPF if ≤ 10 energy percent (E%) was UPF-ingredients. This also means that if the item/dish included non-energy yielding UPF ingredients, the item/dish was classified as non-UPF. If > 10 E% of the item/dish was UPF-ingredients, the dish was disaggregated using recipes from (1) the Swedish food composition table, (2) the traditional Swedish cookbook “Vår kokbok”, or (3) searching online, and choosing any of the top results. Approximately 115 items/dishes were disaggregated. The energy content of the recipe was calculated using the nutrient analysis software Dietist Net Group version 25.04.22 (Kost och Näringsdata AB, Sweden) with the Swedish Food Composition Database version 2024-12-05. The Finnish food composition table (Fineli, version 2020-03-26), Dabas (version 2022-11-13) and nutrient information online was used for four food items that could not be found in the Swedish Food Composition Database. Some considerations were made: (1) In fine baked goods, the fat used was assumed to be butter (non-UPF) unless the participant was categorized as adhering to a vegan diet, then margarine for baking (solid) was assumed instead (UPF), (2) For composite dishes, the cooking fat was not included as a UPF since a smaller amount is used and it would be impossible to know if the participant used butter, oil or margarine, (3) If the recipe included “cream” or “crème fraiche” without further specifications of fat content, it was assumed to be “whipping cream” (40% fat) or crème fraiche with 34% fat unless the participant was categorized as adhering to a vegan diet, then it was assumed to be oat-based fraiche (15% fat). Finally, E% from UPF ingredients was calculated, and this percentage of the food item/dish was classified as UPF and the remaining percent as non-UPF.

For multi-ingredient food items not further specified in product name or the participant’s own note, but which are assumed to be store-bought, e.g., soy sauce, sundried tomatoes, yoghurt, crackers etc., two popular online grocery stores in Sweden (ica.se and coop.se) were searched for the item/dish. If the majority of the 5 most popular products (according to the online grocery stores) of the multi-ingredient food item at each store had ≥ 1 UPF marker, the food item/composite dish was classified as UPF. If the majority had no UPF marker, it was classified as non-UPF. If 5 out of 10 products had UPF-markers, the food item was classified as non-UPF in the main analysis.

Some multi-ingredient food items that possibly, but highly unlikely, could be made from scratch at home were identified as industrially manufactured, e.g., mayonnaise (with/without egg), hot dog- and burger buns, tortilla bread, jam and marmalade, crackers (savory/sweet).

During classification, participants’ notes in the dietary assessment program with details of some foods were considered. If the participant had written in the notes how the dish/bakery product was acquired (homemade/bought at a restaurant/bakery/fast-food chain outlet/grocery store etc.) it was treated as such during classification. Also, which dietary group the participant belonged to was considered. For example, if the participant had been categorized as adhering to a vegan diet, and reported “Cinnamon bun” without further specifications, the bun was considered vegan and thus containing margarine and dairy substitutes.

#### Experimental classification of UPF for sensitivity analyses

In sensitivity analyses, a more experimental approach in classification was undertaken. Here, all uncertain composite dishes and bakery goods were instead assumed to be industrially manufactured. Similarly, as in the main analysis procedure, multi-ingredient food items most likely to be store-bought were searched for at *ica.se* and *coop.se*. If the majority of the 5 most popular products (according to the online grocery stores) at each store had ≥ 1 UPF marker, the food item/composite dish was classified as UPF. If the majority had no UPF marker, it was classified as non-UPF. However, in these analyses, if 5 out of 10 products had UPF-markers, the food item was classified as UPF. Participants’ notes with details of some foods and dietary group were considered also in these analyses.

### Nutrient status: sampling and laboratory analyses of blood

To investigate the participants’ nutrient status in relation to UPF consumption, non-fasted dried blood spots was collected at the study visit, and blood levels of haemoglobin (Hb) and serum transferrin receptor (sTfR) for iron status, homocysteine (tHcy) and methylmalonic acid (MMA) for vitamin B_12_ status, and serum 25-hydroxyvitamin D3 (25-OH-D_3_) for vitamin D status, was analysed. Details regarding collection, analysis and cut-offs have been described previously [26].

There were some missing values: Hb (*n* = 5), sTfR (*n* = 3), MMA (*n* = 45), tHcy (*n* = 5), and 25-OH-D_3_ (*n* = 16). This was due to insufficient blood volume or problems during laboratory analyses. These participants were only removed from the analysis where the value was missing.

### Statistical analysis

Energy intake was the primary outcome of the VeggiSkills-Sweden project and therefore the power calculation was based on this [14]. To detect a difference of 500 kcal between two dietary groups, with a power of 80%, a sample size of 42 participants in each group was needed. To account for dropouts, the goal was to recruit 60 participants per dietary group.

Dietary intake was calculated as the mean intake of the reported days for each participant. Intakes were then reported at the group level. For each dietary group, distributions of the continuous variables were checked using histograms, QQ plots and boxplots. Due to most variables having a skewed distribution, values are presented as median (IQR) and non-parametric tests were performed for all continuous variables. Categorial variables are presented as number (%).

The intake of UPF (using both the conservative and the experimental approach during classification) was calculated as % of total median intake of energy (E%), weight in grams (i.e., total weight of all food and beverages), macro- and micronutrients, whole grain and salt for each participant, and then presented as median (IQR) % of total intake for each dietary group. Additionally, for descriptive purposes only, absolute and relative (to energy intake) intakes of energy, nutrients, whole grain, and salt from UPFs were reported as median (IQR). The primary outcome of this paper was differences in E% UPF between the dietary groups.

Total daily intake (UPF + non-UPF) of energy (kcal), whole grain (g), fiber (g), monounsaturated fatty acids (MUFA), polyunsaturated fatty acids (PUFA) (E%), saturated fatty acids (SFA) (E%), free sugars (E%) and salt (g), i.e., dietary indicators for a healthy/unhealthy food intake available in the dataset, was reported as median (IQR).

To determine possible differences in participant characteristics, UPF intake, and mean total daily intake of dietary indicators for a healthy/unhealthy food intake between the dietary groups, Kruskal Wallis-test with Post hoc comparisons using Bonferroni correction was performed for continuous variables. For categorical variables, χ^2^ test or Fisher-Freeman-Halton Exact Test was performed. Standardized residuals from the χ^2^ tests were inspected to determine which combination of categories contributed most to the significant associations. An absolute value of ≥ 2 or ≤-2 suggested a meaningful deviation from the expected frequency.

Further, associations between E% UPF intake and sex, age, adherence to diet (years), general nutrition knowledge score, BMI, PAL, intake of above-mentioned indicators for a healthy/unhealthy food intake and nutrient status (Hb, sTfR, tHcy, MMA), was analysed in the whole study population. E% UPF (conservative approach) was categorized into quartiles (Q) and Kruskal Wallis-test with Post hoc comparisons using Bonferroni correction was used to test the differences in continuous variables between the quartiles, and χ^2^ tests or Fisher-Freeman-Halton Exact Test was used to test the differences in categorical variables. In addition, standardized residuals from the χ^2^ tests were inspected to determine which combination of categories contributed most to the association. The same associations were also analysed in the separate dietary groups as well as among the participants consuming any of the plant-based diets only, i.e., vegan, lacto-ovo-vegetarian or pescatarian diet. Due to smaller sample sizes in these analyses, E% UPF was instead categorized into tertiles (T).

All statistical analyses were performed in SPSS Statistics Version 30.0.0.0 (IBM Corp., Armonk, NY, United States).

## Results

### Study participants

Of the 244 recruited participants, nine participants were excluded from the study. Six reported consuming a flexitarian diet, one participant reported adherence to a pescatarian diet but also reported consuming meat in the last six months, and two participants (adhering to vegan and lacto-ovo-vegetarian diets) only registered dietary intake for one out of four days. In total, 235 youths, aged 16–24 years old, were included. Of these, 60 consumed a vegan diet, 59 consumed a lacto-ovo-vegetarian diet, 55 consumed a pescatarian diet, and 61 consumed an omnivore diet.

Table 1 describes characteristics of the study population. Median (IQR) age was 22 years (20, 23) and there was no difference between the dietary groups. More than 3/4 of the study population were females, and it did not significantly differ between the dietary groups. Omnivores had a higher median [IQR] BMI compared to lacto-ovo-vegetarians (23 [21, 25] vs. 21 [20, 24], *p* = 0.04), but there were no differences between the other dietary groups, and the proportion of pre-obesity/obesity did not differ between the groups. Years of adherence to the dietary regimen differed between the plant-based dietary groups (*p *=0.020). Standardized residuals suggested that adherence to the diet for ≥10 years was more common among the lacto-ovo-vegetarians (residual = +2.0). In the vegan dietary group, 90% of the participants consumed some kind of supplements, and the proportion of supplement consumers differed between the groups: χ2 (3, n =235) = 22, *p* <0.001. Standardized residuals indicated that the proportion of participants not consuming supplements was lower than expected among the vegans (residual = -2.2) and higher than expected among the omnivores (residual = +3.3).

**Table 1:** Participant characteristics in the cross-sectional study VeggiSkills-Sweden

	All participants (*n* = 235)	Vegan (*n* = 60)	Lacto-ovo-vegetarian (*n* = 59)	Pescatarian (*n* = 55)	Omnivore (*n* = 61)	
	Median (IQR)	Median (IQR)	Median (IQR)	Median (IQR)	Median (IQR)	*p*-value
Age, years	22 (20,, 23)	22 (21,, 23)	22 (20,, 23)	21 (20,, 23)	21 (20,, 23)	**0.048** ^*^
Body mass index, kg/m^2^	22.0 (20.4,, 23.8)	21.9 (20.2,, 23.9)	21.4 (20.1,, 23.5)^a^	21.6 (20.3,, 23.9)	22.5 (21.4,, 24.7)^b^	**0.030** ^*^
	**n (%)**	**n (%)**	**n (%)**	**n (%)**	**n (%)**	
Weight status						0.650^†^
Underweight (BMI < 18.5)	16 (7)	5 (8)	5 (8)	4 (7)	2 (3)	
Normal weight (BMI 18.5–24.9)	176 (75)	43 (72)	47 (80)	41 (75)	45 (74)	
Pre-obesity/Obesity (BMI ≥ 25)	43 (18)	12 (20)	7 (12)	10 (18)	14 (23)	
Female	183 (78)	45 (75)	49 (83)	47 (86)	42 (69)	0.116^‡^
Parental education						
Mother, ≥ 3 y of university education	158 (67)	35 (58)	41 (70)	39 (71)	43 (71)	0.403^‡^
Father, ≥ 3 y of university education	120 (51)	29 (48)	29 (49)	34 (62)	28 (46)	0.326^‡^
Adherence to dietary regimen						**< 0.020** ^†§^
< 1 y	15 (6)	1 (2)	6 (10)	5 (9)	3 (5)	
1–5 y	103 (44)	37 (62)	31 (53)	23 (42)	12 (20)	
> 5–9 y	66 (28)	21 (35)	16 (27)	26 (47)	3 (5)	
≥ 10 y	51 (22)	1 (2)	6 (10)	1 (2)	43 (71)	
Consumption of any dietary supplement(s)^∥^	179 (76)	54 (90)	49 (83)	42 (76)	34 (56)	**< 0.001** ^‡^

Significant *p*-values (*p* < 0.05) are in bold

^*^Differences between the dietary groups were tested using Kruskal Wallis test with Bonferroni post-hoc test to adjust for multiple comparisons. Superscript letters indicate significant differences between groups. Groups sharing the same letter are not significantly different from each other, groups with different letters differ significantly. No superscript letters = no significant differences between any groups

^†^Differences between the dietary groups were tested using Fisher-Freeman-Halton Exact Test

^‡^Differences between the dietary groups were tested using χ^2^-test

^§^Only vegan, lacto-ovo-vegetarian and pescatarian dietary groups were included in the test

^∥^Protein powder (casein/whey) and fortified food items were not included

### UPF intake across the dietary groups

#### Primary outcome: Contribution of UPFs to total intake of energy

The contribution of UPFs to total energy intake (kcal and E%) for both main and sensitivity analyses, i.e. using a conservative vs. experimental approach during UPF classification, is shown in Table 2. Using the conservative approach during classification, UPFs contributed significantly more to total energy intake among the vegan dietary group compared to the other dietary groups (adjusted p-values < 0.05). When using the more experimental approach, there was no statistically significant difference between the dietary groups: *p* = 0.105 (kcal) and 0.055 (E%).

**Table 2:** UPF contribution to energy intake, using two different approaches during classification, in the VeggiSkills-Sweden study

	Conservative approach (main analysis)		Experimental approach (sensitivity analysis)	
	Energy (kcal) Median (IQR)	E% Median (IQR)	Energy (kcal) Median (IQR)	E% Median (IQR)
All participants (*n* = 235)	819 (605, 1091)	37 (30, 47)	1073 (821, 1344)	49 (41, 58)
Vegan (*n* = 60)	1027 (765, 1242)^a^	47 (38, 51)^a^	1187 (849, 1455)	52 (45, 63)
Lacto-ovo-vegetarian (*n* = 59)	785 (576, 1103)^b^	35 (26, 49)^b^	1045 (793, 1302)	47 (39, 59)
Pescatarian (*n* = 55)	776 (591, 876)^b^	35 (31, 42)^b^	1030 (753, 1219)	47 (41, 55)
Omnivore (*n* = 61)	790 (595, 998)^b^	36 (28, 43)^b^	1119 (848, 1376)	48 (39, 56)
p-value*	**< 0.001**	**< 0.001**	0.105	0.055

*E%* Energy percent, *UPF* Ultra-processed food

Significant p-values (*p* < 0.05) are in bold

^*^Differences between the dietary groups were tested using Kruskal Wallis test with Bonferroni post-hoc test to adjust for multiple comparisons. Superscript letters indicate significant differences between groups. Groups sharing the same letter are not significantly different from each other, groups with different letters differ significantly. No superscript letters = no significant differences between any groups

#### Contribution of UPFs to total intake of nutrients and food components

**Fig. 2:**
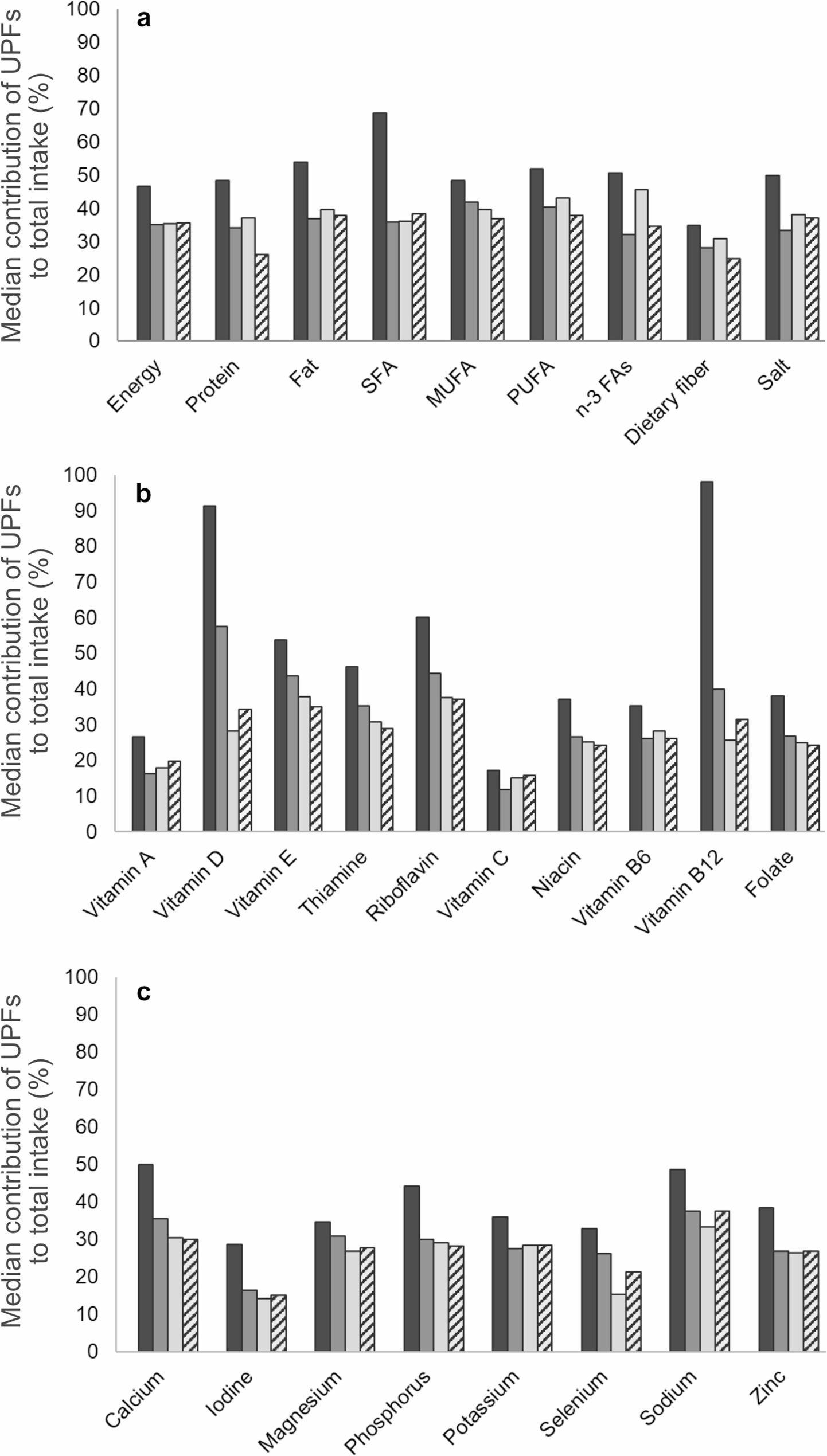
Median percentage contribution (%) of UPFs to energy and nutrient intake in the VeggiSkills-Sweden study. **a** UPF contribution to energy, macronutrients and salt. **b** UPF contribution to vitamins. Vitamin B12: one participant in the vegan dietary group removed from analysis due to incorrect inclusion of supplement in the dataset. **c** UPF contribution to minerals. *Dark grey* Vegan dietary group, n=60, *Medium grey* Lacto-ovo-vegetarian dietary group, n=59, *Light grey* Pescatarian dietary group, n=55, *Striped *Omnivore dietary group, n=61. FA Fatty acids, MUFA Monounsaturated fatty acids, PUFA Polyunsaturated fatty acids, SFA Saturated fatty acids, UPF Ultra-processed food

The contribution of UPFs to total dietary intake (%) is shown in Table 3 and the significant differences between the groups are illustrated in Fig. 2a-c. There was an overall difference in UPF contribution between the dietary groups for all macro- and micronutrients except carbohydrates, free sugars and vitamin K, and for the food components whole grain and salt (Table 3). With the Bonferroni post-hoc test, the difference between the dietary groups was no longer statistically significant for iron and whole grain.

**Table 3:** Percentage contribution (%) of UPFs (conservative approach) to total intake in the VeggiSkills-Sweden study

	All participants (n=235)	Vegan (n=60)	Lacto-ovo-vegetarian (n=59)	Pescatarian (n=55)	Omnivore (n=61)	
	Median (IQR)	Median (IQR)	Median (IQR)	Median (IQR)	Median (IQR)	*p*-value
Weight of food and beverages	23.9 (16.5, 32.6)	27.6 (19.1, 39.6)	23.4 (14.8, 34.1)	23.6 (17.6, 29.4)	22.1 (15.9, 31.2)	0.068
Energy	37.4 (29.7, 46.7)	46.7 (37.8, 51.4)^a^	35.4 (26.3, 48.5)^b^	35.1 (31.2, 42.3)^b^	35.6 (28.5, 43.3)^b^	<**0.001**
Protein	35.6 (23.6, 48.1)	48.4 (37.0, 57.6)^a^	37.0 (24.2, 48.3)^b^	34.1 (21.7, 40.5)^bc^	26.1 (19.0, 35.8)^c^	<**0.001**
Fat	41.2 (31.9, 52.8)	53.9 (43.3, 60.9)^a^	39.5 (28.1, 50.4)^b^	36.9 (31.2, 47.0)^b^	37.9 (26.5, 44.4)^b^	<**0.001**
SFA	42.8 (30.2, 57.6)	68.7 (55.2, 76.0)^a^	36.1 (24.1, 54.4)^b^	35.8 (27.9, 44.2)^b^	38.3 (23.4, 46.3)^b^	<**0.001**
MUFA	40.9 (31.2, 51.6)	48.3 (35.1, 57.7)^a^	39.5 (28.1, 51.7)	42.0 (28.5, 49.4)	36.9 (29.5, 48.0)^b^	**0.004**
PUFA	42.1 (31.6, 55.8)	51.9 (38.5, 61.6)^a^	43.0 (28.7, 57.6)	40.3 (28.8, 48.4)^b^	37.9 (25.5, 50.8)^b^	<**0.001**
n-3 fatty acids^†^	40.7 (26.7, 55.6)	50.7 (37.3, 67.1)^a^	45.6 (27.6, 55.7)	32.0 (23.6, 48.6)^b^	34.7 (18.5, 49.8)^b^	<**0.001**
Carbohydrates	37.8 (27.2, 46.3)	42.2 (28.8, 49.2)	34.6 (24.4, 45.5)	36.9 (28.8, 44.7)	37.7 (25.7, 45.8)	0.204
Dietary fiber	30.8 (20.0, 41.3)	34.9 (28.2, 42.1)^a^	30.8 (19.2, 42.0)	28.1 (20.0, 39.1)	24.7 (16.1, 40.5)^b^	**0.013**
Whole grain^‡^	16.9 (0.4, 47.6)	28.8 (7.8, 52.9)	16.5 (4.4, 49.7)	8.1 (0.0, 43.5)	12.4 (0.0, 44.8)	**0.048**
Free sugars^§^	72.1 (51.6, 87.8)	72.0 (47.1, 87.8)	67.8 (42.2, 84.2)	77.7 (59.1, 89.7)	71.7 (52.6, 87.5)	0.320
Salt	38.4 (29.9, 52.3)	50.0 (38.9, 62.9)^a^	38.0 (29.2, 53.8)^b^	33.4 (26.0, 43.9)^b^	37.2 (27.3, 49.9)^b^	<**0.001**
Vitamin A	19.1 (11.3, 34.2)	26.5 (15.5, 43.3)^a^	16.3 (9.9, 31.8)^b^	17.9 (11.9, 28.1)	19.7 (10.6, 34.4)	**0.023**
Vitamin D	49.3 (24.0, 84.0)	91.2 (81.0, 99.0)^a^	57.5 (30.1, 79.7)^b^	28.1 (10.9, 55.9)^c^	34.2 (14.1, 48.3)^cd^	<**0.001**
Vitamin E	42.4 (29.7, 55.6)	53.7 (41.5, 62.1)^a^	43.6 (28.6, 56.3)^b^	37.9 (26.1, 49.2)^b^	34.9 (25.9, 50.5)^b^	<**0.001**
Vitamin K	13.9 (6.4, 25.4)	13.9 (7.6, 25.7)	13.0 (6.0, 23.4)	17.6 (6.4, 26.5)	14.3 (6.7, 24.7)	0.928
Thiamine	36.7 (23.4, 48.6)	46.4 (38.2, 52.6)^a^	35.2 (22.9, 51.5)	30.8 (20.6, 48.6)^b^	29.0 (19.9, 41.6)^b^	<**0.001**
Riboflavin	45.3 (30.6, 60.5)	60.1 (50.2, 69.1)^a^	44.5 (29.7, 57.5)^b^	37.7 (29.3, 50.8)^b^	37.0 (23.3, 47.7)^b^	<**0.001**
Vitamin C	15.0 (6.6, 28.8)	17.1 (9.7, 33.5)^a^	11.8 (4.7, 20.6)^b^	15.0 (6.6, 27.6)	15.8 (5.9, 37.9)	**0.048**
Niacin	27.7 (19.1, 41.8)	37.1 (28.1, 49.0)^a^	26.7 (17.2, 40.5)^b^	25.1 (18.5, 39.1)^b^	24.3 (16.4, 37.9)^b^	<**0.001**
Vitamin B_6_	28.8 (19.8, 42.5)	35.2 (26.5, 50.7)^a^	26.1 (20.4, 42.5)	28.3 (18.9, 41.4)	26.1 (17.3, 35.6)^b^	**0.016**
Vitamin B_12_	46.2 (22.6, 80.7)^∥^	98.0 (90.5, 100)^∥a^	39.9 (25.0, 63.8)^b^	25.7 (12.0, 47.0)^b^	31.5 (16.8, 48.2)^b^	<**0.001**^∥^
Folate	27.4 (20.1, 41.9)	38.0 (24.9, 45.1)^a^	26.9 (20.0, 44.8)	24.9 (20.2, 38.6)^b^	24.2 (16.9, 38.3)^b^	**0.001**
Iodine	18.0 (9.8, 30.6)	28.7 (15.1, 41.3)^a^	16.3 (9.8, 30.4)^b^	14.2 (9.3, 21.4)^b^	15.1 (7.2, 26.3)^b^	<**0.001**
Phosphorus	31.5 (24.5, 44.2)	44.1 (33.0, 53.0)^a^	29.9 (22.2, 45.8)^b^	29.1 (23.9, 38.2)^b^	28.2 (20.3, 35.0)^b^	<**0.001**
Iron	34.5 (24.6, 47.5)	44.0 (27.8, 50.8)	36.8 (23.9, 51.7)	32.5 (23.7, 41.8)	31.1 (22.5, 42.1)	**0.047**
Calcium	36.3 (22.8, 49.8)	50.0 (38.9, 62.9)^a^	35.4 (22.8, 49.8)^b^	30.3 (20.6, 42.2)^b^	30.0 (18.6, 40.8)^b^	<**0.001**
Potassium	29.8 (21.7, 38.9)	35.9 (22.8, 45.3)^a^	27.6 (20.1, 42.0)	28.4 (22.8, 34.5)	28.5 (17.9, 36.1)^b^	**0.028**
Magnesium	28.5 (21.4, 40.4)	34.6 (25.0, 44.1)^a^	30.7 (20.9, 44.6)	26.9 (21.5, 38.0)	27.6 (18.0, 34.6)^b^	**0.030**
Sodium	38.3 (29.9, 51.5)	48.7 (38.3, 61.1)^a^	37.4 (28.8, 51.5)^b^	33.2 (25.4, 43.1)^b^	37.5 (27.2, 49.7)^b^	**<0.001**
Selenium	23.5 (13.8, 36.9)	32.9 (22.0, 47.7)^a^	26.1 (14.5, 40.9)^ab^	15.4 (10.6, 25.4)^c^	21.2 (12.8, 29.7)^bc^	<**0.001**
Zinc	28.8 (20.6, 42.1)	38.4 (28.5, 46.7)^a^	26.9 (16.4, 44.7)^b^	26.4 (20.3, 40.7)^b^	26.9 (19.5, 33.1)^b^	<**0.001**

UPF Ultra-processed food

Significant *p*-values (*p*<0.05) are bold

^*^Differences between the dietary groups were tested using Kruskal Wallis test with Bonferroni post-hoc test to adjust for multiple comparisons. Superscript letters indicate significant differences between groups. Groups sharing the same letter are not significantly different from each other, groups with different letters differ significantly. No superscript letters = no significant differences between any groups

†The sum of alpha-linolenic acid, linoleic acid, eicosapentaenoic acid, docohexaenoic acid, and docosapentaenoic acid

^‡^Missing data due to no total intake of whole grain (total n=5, vegan n=2, lacto-ovo-vegetarian n=2, pescetarian n=1)

^§^Missing data due to no total intake of free sugars (omnivore n=1)

^∥^n=234. One participant removed from analyses due to incorrect inclusion of vitamin B12 supplement in the dataset

The vegan dietary group had the highest contribution from UPF to total intake of energy, protein, total fat, SFA, vitamin D, vitamin E, riboflavin, niacin, vitamin B_12_, iodine, phosphorus, calcium, sodium, zinc, and salt. The contribution of UPF to total intake of protein and vitamin D were significantly higher for the lacto-ovo-vegetarian dietary group compared to the pescetarian and/or omnivore dietary group. The vegan dietary group had a significantly higher contribution from UPF to total intake of MUFA, PUFA, n-3 fatty acids, thiamine, vitamin B_6_, folate, potassium, magnesium, selenium and fiber, compared to the pescetarian and/or omnivore dietary group. Also, the lacto-ovo-vegetarian dietary group had a higher contribution from UPF to total intake of selenium compared to the pescatarian dietary group. The vegan dietary group had a higher contribution from UPF to total intake of vitamin A and vitamin C compared to the lacto-ovo-vegetarian dietary group.

Absolute and relative intake of energy, nutrients, whole grain, and salt from UPFs is reported in Suplemental Table 1.

The UPF intake in the dietary groups using an experimental approach during classification are shown in Supplemental Table 2. Compared to the other dietary groups, vegans had higher contributions of intake of several nutrients from UPF, although not for as many nutrients as in the main analysis which uses a conservative approach.

### Dietary indicators for a healthy/unhealthy food intake

Table 4 displays daily intakes of energy, whole grain, fiber, MUFA, PUFA, SFA, free sugars and salt expressed as median (IQR) values. Vegans had a higher fiber (g/d) intake (*p* < 0.010), a higher PUFA (E%) intake (*p* < 0.001), and a lower SFA (E%) intake (*p* < 0.001), than lacto-ovo-vegetarians, pescatarians and omnivores. Further, lacto-ovo-vegetarians had a higher intake of PUFA (E%) and a lower intake of SFA (E%) than omnivores (*p* < 0.05).

**Table 4:** Dietary indicators for a healthy/unhealthy food intake in 16-24-year-olds participating in the VeggiSkills-Sweden study

	All participants (*n* = 235)	Vegan (*n* = 60)	Lacto-ovo-vegetarian (*n* = 59)	Pescatarian (*n* = 55)	Omnivore (*n* = 61)	
	Median (IQR)	Median (IQR)	Median (IQR)	Median (IQR)	Median (IQR)	*p*-value^*^
Energy, kcal	2196 (1893, 2498)	2228 (1933, 2470)	2088 (1788, 2549)	2104 (1847, 2365)	2346 (1961, 2706)	0.056
Whole grain, g	39 (20, 62)	36 (21, 61)	38 (18, 59)	35 (13, 63)	40 (23, 79)	0.516
Dietary fiber, g	28 (22, 35)	35 (28, 40)^a^	27 (24, 33)^b^	24 (20, 31)^b^	25 (19, 32)^b^	**< 0.001**
Monounsaturated fatty acids, E%	15 (13, 17)	15 (13, 18)	15 (13, 17)	15 (14, 17)	15 (13, 18)	0.934
Polyunsaturated fatty acids, E%	7 (6, 8)	8 (8, 10)^a^	7 (6, 8)^b^	7 (6, 8)^bc^	6 (5, 7)^c^	**< 0.001**
Saturated fatty acids, E%	11 (9, 14)	8 (6, 9)^a^	11 (9, 14)^b^	12 (11, 15)^bc^	13 (12, 16)^c^	**< 0.001**
Free sugars, E%	4 (2, 6)	3 (2, 5)	4 (2, 5)	4 (3, 6)	3 (1, 6)	0.071
Salt, g	9 (7, 11)	8 (6, 12)	9 (6, 11)	9 (7, 10)	9 (7, 11)	0.365

Significant *p*-values (*p* < 0.05) are in bold

^*^Differences between the dietary groups were tested using Kruskal Wallis test with Bonferroni post-hoc test to adjust for multiple comparisons. Superscript letters indicate significant differences between groups. Groups sharing the same letter are not significantly different from each other, groups with different letters differ significantly. No superscript letters = no significant differences between any groups

### Associations between UPF intake and participant characteristics, and dietary indicators for a healthy/unhealthy food intake

**Fig. 3:**
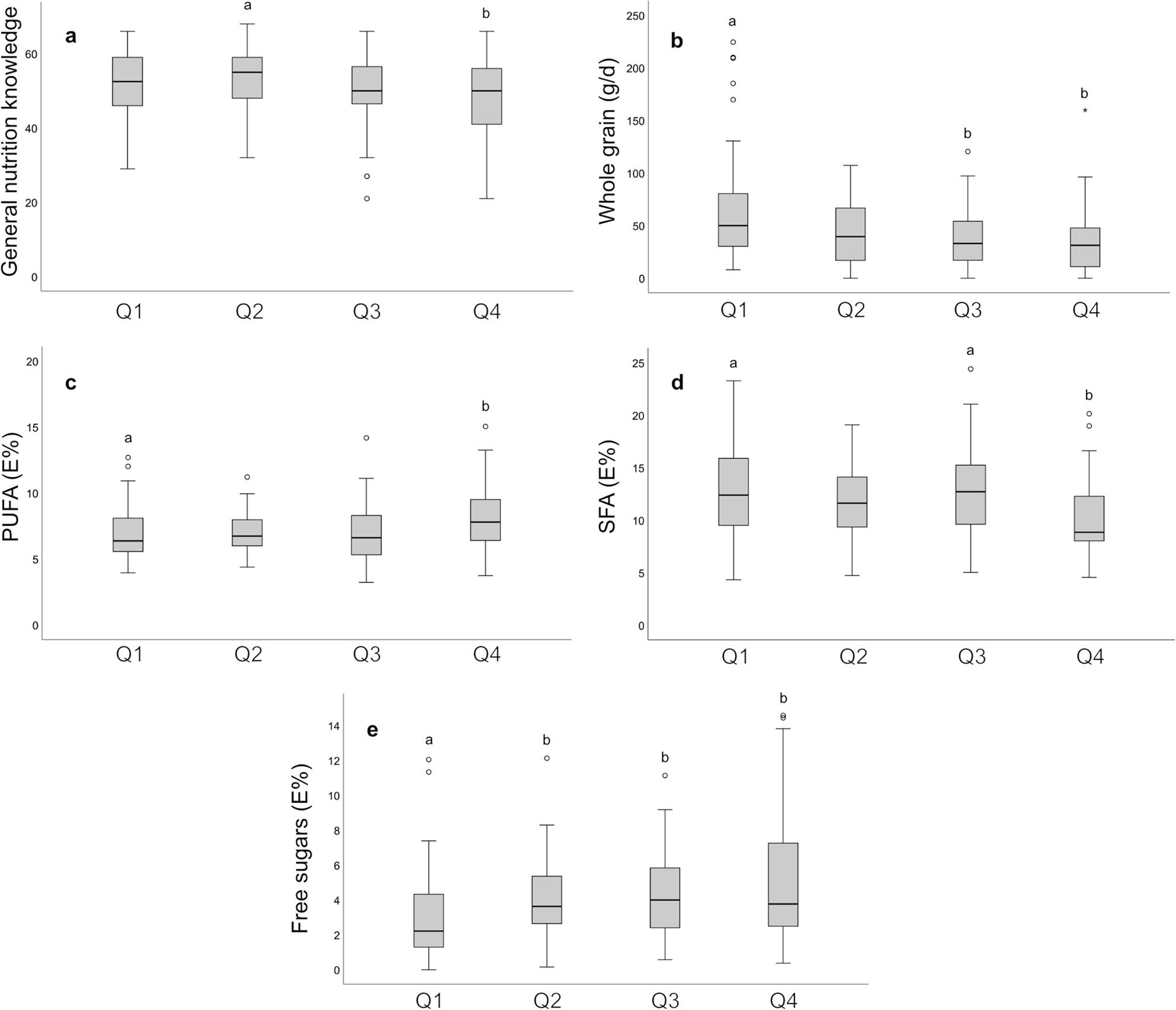
GNK score and nutrient intake across quartiles of UPF intake in the VeggiSkills-Sweden study. **a** Boxplots showing general nutrition knowledge score across quartiles of UPF intake. **b**-**e** Boxplots showing daily intake of dietary indicators for a healthy/unhealthy food intake across quartiles of UPF intake. Superscript letters indicate significant differences between dietary groups (p<0.05). Groups sharing the same letter are not significantly different from each other, groups with different letters differ significantly. E% Energy percent, GNK General nutrition knowledge, PUFA Polyunsaturated fatty acids, Q Quartile, SFA Saturated fatty acids, UPF Ultra-processed food

When dividing the whole study population (*n* = 235) into quartiles based on E% of UPF intake, there were no associations between age, sex, adherence to dietary regimen (in years), PAL or BMI, and UPF intake (Table 5). The general nutrition knowledge was significantly lower in Q4 compared to Q2; median (IQR) score 50 (41, 56) vs. 55 (48, 59), and the proportion of participants with low, moderate and high level of general nutrition knowledge differed between the quartiles: χ^2^ (6, *n* = 235) = 14, *p* = 0.033, with the proportion of participants with a low level of general nutrition knowledge being higher in Q4 (standardized residuals = + 2.2). Figure 3a illustrates the differences in nutrition knowledge between the quartiles. General nutrition knowledge did not differ by quartile E% UPF when including only participants consuming plant-based diets (Supplemental Table 3). Further, among the participants consuming plant-based diets, E% UPF intake was negatively associated with duration of dietary adherence: χ^2^ (3, *n* = 174) = 7.9, *p* = 0.048 (Supplemental Table 3). The standardized residuals did, however, not indicate strong deviations from expected values in any cell.

**Table 5:** Associations between UPF intake and participant characteristics and nutrient intake in in the VeggiSkills-Sweden study

	E% UPF intake (range)				
All participants, n=235	Q1 (2.6-29.6 E%)	Q2 (29.7-37.3 E%)	Q3 (37.5-46.6 E%)	Q4 (46.7-81.9 E%)	*p*-value
Sex					0.729^*^
Female	47 (81)	43 (16)	46 (78)	47 (80)	
Male	11 (19)	16 (27)	13 (22)	12 (20)	
Age, years	22 (20, 23)	21 (20, 23)	22 (20, 23)	22 (21, 23)	0.802^†^
Adherence to dietary regimen					0.168^*^
<5 years	22 (38)	30 (51)	33 (56)	33 (56)	
≥5 years	36 (62)	29 (49)	26 (44)	26 (44)	
General nutrition knowledge score	52 (46, 59)	55 (48, 59)^a^	50 (46, 57)	50 (41, 56)^b^	**0.023** ^†^
Level of general nutrition knowledge					**0.033** ^*^
Low level	6 (10)	5 (9)	10 (17)	16 (27)	
Moderate level	32 (55)	33 (56)	36 (61)	33 (56)	
High level	20 (35)	21 (36)	13 (22)	10 (17)	
Body mass index, kg/m^2^	21.5 (20.1, 23.6)	21.8 (20.4, 24.1)	22.3 (21.1, 23.7)	22.2 (20.3, 23.9)	0.295^†^
Weight status					0.967^‡^
Underweight (BMI <18.5)	4 (7)	4 (7)	4 (7)	4 (7)	
Normal weight (BMI 18.5-24.9)	46 (79)	42 (71)	44 (75)	44 (75)	
Pre-obesity/Obesity (BMI ≥25)	8 (14)	13 (22)	11 (19)	11 (19)	
Physical Activity Level					0.251^*^
1.4 or 1.55	15 (26)	11 (19)	14 (24)	20 (34)	
1.7	23 (40)	22 (37)	29 (49)	24 (41)	
1.8 or 2.0	20 (34)	26 (44)	16 (27)	15 (25)	
Energy intake, kcal	2242 (1828, 2514)	2242 (1911, 2487)	2196 (1914, 2692)	2118 (1928, 2446)	0.870^†^
Whole grain intake, g	50 (30, 83)^a^	40 (16, 68)	33 (17, 55)^b^	31 (9, 48)^b^	**0.002** ^†^
Dietary fiber intake, g	28 (22, 36)	29 (23, 33)	27 (21, 35)	27 (22, 35)	0.802^†^
Monounsaturated fatty acids intake, E%	15 (13, 17)	15 (13, 17)	15 (13, 17)	15 (13, 18)	0.571^†^
Polyunsaturated fatty acids intake, E%	6 (6, 8)^a^	7 (6, 8)	7 (5, 8)	8 (6, 10)^b^	**0.012** ^†^
Saturated fatty acids intake, E%	12 (9, 16)^a^	12 (9,14)	12 (10, 15)^a^	9 (8, 12)^b^	<**0.001**^†^
Free sugars intake, E%	2 (1, 4)^a^	4 (3, 5)^b^	4 (2, 6)^b^	4 (2, 7)^b^	**0.002** ^†^
Salt intake, g	9 (6, 11)	9 (7, 12)	9 (7, 10)	8 (7, 11)	0.609^†^

*UPF *Ultra-processed food, *Q* Quartile

Values are median (IQR) for continuous data and n (%) for categorical data. Significant p-values (*p*<0.05) are in bold

^*^Differences between the quartiles were tested using χ2 test

^†^Differences between the quartiles were tested using Kruskal Wallis test with Bonferroni post-hoc test to adjust for multiple comparisons. Superscript letters indicate significant differences between groups. Groups sharing the same letter are not significantly different from each other, groups with different letters differ significantly. No superscript letters = no significant differences between any groups

^‡^Differences between the quartiles were tested using Fisher-Freeman-Halton Exact test

In the total sample (*n* = 235) there were no differences in total (UPF + non-UPF) intake of energy, fiber, MUFA or salt between the quartiles of E% UPF. The median total intake of whole grain (g) was higher in Q1 compared to Q3 and Q4, *p* < 0.05 (Table 5; Fig. 3b), and the median total intake of PUFA (E%) was higher in Q4 compared to Q1, *p* = 0.014 (Table 5; Fig. 3c). The median total intake of SFA (E%) was lower in Q4 compared to Q3 and Q1, *p* < 0.05 (Table 5; Fig. 3d), and median total intake of free sugars (E%) was lower in Q1 compared to all other quartiles, *p* < 0.05 (Table 5; Fig. 3e). Including only participants consuming plant-based diets, the associations were overall similar except for free sugars which did not differ between the quartiles at all (Supplemental Table 3). 

### Associations between UPF intake and participant characteristics, and dietary indicators for a healthy/unhealthy food intake, stratified by dietary regimen

In the vegan dietary group, the median (IQR) total intake of free sugars (E%) was lower in T1 compared to T3: 2 [1, 3] vs. 5 [2, 9], *p* < 0.05 (Table 6). There were no associations between UPF intake and dietary indicators of a healthy/unhealthy food intake or age, sex, adherence to dietary regimen in years, general nutrition knowledge or PAL. In the omnivore dietary group, the general nutrition knowledge was lower in T3 compared to T1 and T2 (*p* < 0.05) and there was a significant difference between the tertiles regarding level of general nutrition knowledge (*p* = 0.017) (Table 6). Furthermore, among omnivores, the SFA intake (E%) was higher in T3 compared to T2 (*p* = 0.021) and the intake of free sugars (E%) was lower in T1 compared to both T2 and T3 (*p* < 0.05). Finally, the salt intake was higher in T2 compared to T3 among omnivores (*p* = 0.023).

**Table 6:** Associations between UPF intake and participant characteristics and nutrient intake in young vegans and omnivores

	E% UPF intake (range)			
Vegan dietary group, n=60	T1 (11.8-40.6)	T2 (41.8-48.4)	T3 (48.9-81.9)	*p*-value
Sex				0.155^*^
Female	12 (60)	16 (80)	17 (85)	
Male	8 (40)	4 (20)	3 (20)	
Age, years	23 (22, 24)	22 (21, 23)	22 (20, 23)	0.311^†^
Adherence to dietary regimen				0.256^*^
<5 years	13 (65)	15 (75)	10 (50)	
≥5 years	7 (35)	5 (25)	10 (50)	
General nutrition knowledge score	52 (43, 56)	54 (48, 61)	54 (44, 58)	0.477^†^
General nutrition knowledge level				0.900^*^
Low level	4 (20)	2 (10)	3 (15)	
Moderate level	12 (60)	12 (60)	12 (60)	
High level	4 (20)	6 (30)	5 (25)	
Body mass index, kg/m^2^	21.5 (20.3, 23.9)	21.9 (19.7, 24.3)	21.9 (20.1, 23.8)	0.970^†^
Body mass index category				1.000^‡^
Underweight (Body mass index <18.5)	2 (10)	2 (10)	1 (5)	
Normal weight (Body mass index 18.5-24.9)	14 (70)	14 (70)	15 (75)	
Pre-obesity/Obesity (Body mass index ≥25)	4 (20)	4 (20)	4 (20)	
Physical Activity Level				0.087^*^
1.4 or 1.55	4 (20)	7 (35)	9 (45)	
1.7	10 (50)	11 (55)	4 (20)	
1.8 or 2.0	6 (30)	2 (10)	7 (35)	
Energy intake, kcal	2272 (1815, 2524)	2239 (2002, 2579)	2172 (1958, 2415)	0.666^†^
Whole grain intake, g	51 (27, 69)	32 (22, 40)	35 (9, 61)	0.143^†^
Dietary fiber intake, g	36 (28, 46)	36 (32, 40)	31 (24, 37)	0.060^†^
Monounsaturated fatty acids intake, E%	14 (12, 17)	15 (14, 17)	14 (13, 20)	0.526^†^
Polyunsaturated fatty acids intake, E%	9 (7, 11)	8 (8, 10)	10 (8, 11)	0.098^†^
Saturated fatty acids intake, E%	7 (6, 9)	8 (7, 9)	8 (7, 11)	0.393^†^
Free sugars intake, E%	2 (1, 3)^a^	4 (2, 6)	5 (2, 9)^b^	**0.005** ^†^
Salt intake, g	7 (6, 10)	10 (7, 14)	8 (6,12)	0.061^†^
**Omnivore dietary group, n=60**	**T1 (2.6-29.8)**	**T2 (30.6-41.0)**	**T3 (41.7-72.5)**	
Sex				0.764^*^
Female	15 (75)	14 (67)	13 (65)	
Male	5 (25)	7 (33)	7 (100)	
Age, years	21 (19, 23)	21 (20, 22)	22 (20, 23)	0.559^†^
Adherence to dietary regimen				1.000^‡^
<5 years	5 (25)	5 (24)	5 (25)	
≥5 years	15 (75)	16 (76)	15 (75)	
General nutrition knowledge score	54 (48, 62)^a^	56 (49, 59)^a^	48 (35, 55)^b^	**0.009** ^†^
General nutrition knowledge level				**0.017** ^‡^
Low level	1 (5)	1 (5)	7 (35)	
Moderate level	11 (55)	11 (52)	11 (55)	
High level	8 (40)	9 (43)	2 (10)	
Body mass index, kg/m^2^	22 (21, 24)	24 (22, 25)	23 (21, 24)	0.113^†^
Body mass index category				0.573^‡^
Underweight (Body mass index <18.5)	1 (5)	0 (0)	1 (5)	
Normal weight (Body mass index 18.5-24.9)	16 (80)	14 (67)	15 (75)	
Pre-obesity/Obesity (Body mass index ≥25)	3 (15)	7 (33)	4 (20)	
Physical Activity Level				0.946^‡^
1.4 or 1.55	3 (15)	4 (19)	3 (15)	
1.7	7 (35)	5 (24)	7 (35)	
1.8 or 2.0	10 (50)	12 (57)	10 (50)	
Energy intake, kcal	2334 (1917, 2844)	2355 (1982, 2686)	2334 (1845, 2772)	0.954^†^
Whole grain intake, g	56 (32, 98)	39 (18, 74)	35 (14, 53)	0.117^†^
Dietary fiber intake, g	27 (20, 34)	25 (19, 32)	21 (15, 26)	0.067^†^
Monounsaturated fatty acids intake, E%	15 (14, 19)	15 (13, 18)	15 (13, 19)	0.877^†^
Polyunsaturated fatty acids intake, E%	6 (6, 7)	6 (5, 7)	5 (4, 7)	0.104^†^
Saturated fatty acids intake, E%	14 (11, 17)	13 (10, 14)^a^	15 (13, 16)^b^	**0.024** ^†^
Free sugars intake, E%	1 (1, 3)^a^	5 (3, 6)^b^	4 (2, 5)^b^	0.002^†^
Salt intake, g	10 (7, 12)	11 (9, 14)^a^	8 (7, 9)^b^	**0.026** ^†^

*E*% Energy percent, *T* Tertile, *UPF* Ultra-processed food

Values are median (IQR) for continuous data and no. (%) for categorical data. Significant *p*-values (*p* <0.05) are in bold. Data from the VeggiSkills-Sweden study

^*^Differences between the quartiles were tested using χ^2^ test

^†^Differences between the quartiles were tested using Kruskal Wallis test with Bonferroni post-hoc test to adjust for multiple comparisons. Superscript letters indicate significant differences between groups. Groups sharing the same letter are not significantly different from each other, groups with different letters differ significantly. No superscript letters = no significant differences between any groups

^‡^Differences between the tertiles were tested using Fisher-Freeman-Halton Exact test

Data for lacto-ovo-vegetarians and pescatarians is available in Supplementary Table 4

Associations between UPF intake and participant characteristics and indicators of a healthy/unhealthy food intake in the lacto-ovo-vegetarian and pescatarian dietary groups are displayed in Supplemental Table 4. There were no significant associations in the lacto-ovo-vegetarian dietary group. As in the whole study population, UPF intake was negatively associated with general nutrition knowledge and whole grain in the pescatarian dietary group. Among the pescatarians, a higher UPF intake was also associated with a lower fiber intake, *p* = 0.028 (Supplemental Table 4). 

### Associations between UPF intake and nutrient status

There were no associations between E% UPF intake and nutrient status, except for Hb level among the vegans. Hb was higher in T1 compared to T2 among the vegans (*p* = 0.025), but it did not differ from T3 (Supplemental Tables 5 and Supplemental Fig. 1). There were no associations between UPF intake and proportion at risk of insufficiency for iron, vitamin B_12_, or vitamin D (Supplemental Table 6).

## Discussion

This cross-sectional study including a youth population of 16–24-year-olds in Sweden, found that among all participants, higher UPF intake was associated with a lower intake of whole grain and a higher intake of free sugars. Surprisingly, a lower UPF intake was associated with higher SFA and lower PUFA intake. Furthermore, youth adhering to a vegan diet obtained more energy, nutrients (including nutrients of concern in plant-based diets) and salt from UPFs compared to youth adhering to a lacto-ovo-vegetarian, pescatarian or omnivore diet. Despite this, vegans did not have a higher energy intake or BMI, and they did not have a higher total intake of salt or free sugars. Also, vegans consumed more fiber and PUFA, and less SFA compared to those adhering to the other dietary regimes. Thus, despite the higher UPF consumption, vegans did not seem to eat unhealthier than those consuming less strict plant-based or omnivore diets.

Some of our results are in line with previous research. For example, Gehring et al., who investigated UPF consumption in a French adult population of > 20,000 individuals with a mean age of 56 years, concluded that vegans and vegetarians obtained more energy from UPF compared to meat eaters [27]. Other studies have obtained slightly different results. In a large British cohort study including 40–69-year-olds, a higher UPF intake was observed among vegans and lacto-/ovo-vegetarians compared to meat eaters when, similar to our study, including only participants with at least two 24 h-recalls [28]. However, in the main analysis, including all participants regardless of number of recalls, there was no difference between vegans and meat eaters. Dos Santos et al. did not include meat-eaters but found, in contrast to the present study, that among Brazilian adults, lacto- and ovo-vegetarians consumed more UPFs (times/day) compared to vegans [29], and, finally, Fedde et al. did not see any differences in E% UPF at all between vegan, vegetarian and omnivore German first-year college students [30].

Reported contribution of UPFs to total energy intake among individuals following plant-based diets has also varied. Similar to our study, E% UPF intake was 46% among the vegan college students in Germany [30] while in the French study it was slightly lower; 40 E% [27]. This difference could be partly due to the differences in mean/median age of participants between these studies. Younger populations tend to consume more UPFs [31, 32] which was also confirmed among the pescatarians, vegetarians and vegans in the French study [27]. Moreover, the dietary data in both our Swedish study and the German study was collected more than ten years later than the data in the French study. The plant-based market has evolved tremendously during recent years offering several different kinds of plant-based milk and PBMAs, both categorized as UPFs [3–6]. This could be a reason for the higher contribution of UPF to energy intake in recent studies, especially among vegans. On the other hand, Chang et al. reported in a British study, a slightly higher UPF intake (50 E%) among the middle aged vegan population with data collected 8–13 y earlier [28]. As indicated by the present study, higher nutrition knowledge seems to be associated with lower UPF intake. Because of the recruitment method (convenience and snowball sampling), our study population was most likely more interested in diet and health and thus more likely to be more well-informed about that than in the other study including participants identified from a patient registry in the UK Biobank study. This could, in part, explain the differences.

The French study [27] showed associations between a higher UPF consumption and shorter duration of adherence to the plant-based diets (i.e., vegan, vegetarian and pescatarian). Furthermore, BMI ≥ 30, low physical activity and being a woman was associated with higher UPF consumption in these diets. Also a Brazilian study showed similar associations; increased UPF consumption associated with increased overweight probability and a shorter adherence to the diets was associated with higher UPF consumption [29]. These results were not confirmed by the present study where only a weak association between duration of adherence to the plant-based diets and no associations between BMI, physical activity, or sex and E% UPF intake was found. Similar to our findings, no associations between UPF intake and BMI were found among German college students consuming plant-based and omnivore diets [30]. Lack of power could be a reason for not detecting any significant associations. In the present study, the power calculation was performed for VeggiSkills-Sweden’s primary outcome energy intake and in the German study even a smaller number of participants (*n* = 142) was participating. Also, different statistical methods were used to investigate associations.

In the German study, it was concluded that the consumed UPFs contained less micronutrients compared to the Nova group 1–3 foods [30]. However, in the vegan and vegetarian diets, the contribution of UPFs to critical nutrients was larger than the contribution from other Nova groups. In the present study, almost all vitamin D and vitamin B_12_ were obtained from UPFs among the youth consuming a vegan diet. However, no association was found between UPF intake and nutrient status across the sample or within each dietary group. The only biomarker with significant differences between the ranges of UPF intake was Hb, and this was only among the vegans. To our knowledge, this is the first study investigating associations between UPF consumption and iron, vitamin B_12_, and vitamin D status among a youth population.

However, a high UPF consumption have been associated with elevated tHcy levels (indicating inadequate vitamin B_12_ status) among women in the periconceptional period [33]. Further, in an adult Brazilian population, high UPF intake was associated with vitamin D deficiency [34]. In the present study, vegans in the second tertile of E% UPF intake had lower Hb level compared to those with the lowest UPF intake (first tertile), but it did not differ to those consuming the most UPFs (third tertile). The bioavailability of iron in PBMAs is very low [35] which could be one possible explanation for the lower Hb status in the second tertile. Still, no participant had levels corresponding to anaemia or iron insufficiency. Thus, this significant finding is unlikely to be of clinical importance and should be interpreted cautiously.

In Sweden, ultra-processed plant-based milk is fortified with vitamin D and most often also with B-vitamins and iodine [36]. Although not as common, some ultra-processed PBMAs are also fortified with varying vitamins and minerals such as iron and vitamin B_12_ [37]. It could therefore be expected that vegans with high intake of UPF would have higher blood levels of biomarkers for these nutrients. However, the amount of supplement users was high in this study population, especially among vegans, which could be a reason for the homogenic nutrient status over the E% UPF tertiles.

Our results slightly differed depending on the UPF classification approach used. In the experimental approach, only a trend towards difference in E% UPF between the dietary groups was observed. Meanwhile, in the conservative approach, a clear significant difference in E% UPF was found between the dietary groups. These observed differences underscore the importance of consensus regarding classification of UPF to enable fair comparisons between studies as well as the importance of developing dietary assessment methods that provide sufficient details to obtain information on UPF consumption.

Some countries, such as Brazil [38] and France [39], discourage consumption of UPFs in their dietary guidelines. Even though many scientific results points towards a higher energy intake and weight gain as well as a higher risk for non-communicable diseases with increased intake of UPFs, the NNR 2023 do not include specific recommendations on UPF intake [1, 40]. Not including this was due to their view that the ultra-processed food categorization did not add to the already existing classifications of foods and recommendations. UPFs are defined without considering nutrient content and include also nutrient dense food items such as whole grain bread. Considering the results of this study and the consensus on encouraging more plant-forward diets, the restrictive approach to the term ‘ultra-processed foods’ and carefulness in providing specific recommendations should be retained.

### Limitations and strengths

The VeggiSkills-Sweden study was not designed to specifically measure UPF intake. Thus, instructions on how to report foods did not emphasize reporting brand name or where the food was prepared. We tried to overcome this by having both a conservative and experimental approach during classification. Unfortunately, previously published and unpublished ´best practices´-protocols on how to apply the Nova classification system could not be strictly adhered to; for example, due to resource constraints, the classification was performed by only one researcher. However, some uncertainties were discussed with another researcher in the field. Other deviations from the protocols are described in Supplementary Text 1. Also, the Nova classification system has limitations, e.g., it does not include any nutritional aspects. Still, this is the most used system to classify food items in terms of the extent of industrial processing and by using this system, our results can more appropriately be compared to others.

Misreporting when registering food intake is common. It has been suggested that unhealthy foods rich in energy such as fast food, candy, sodas, pastries – i.e. often UPFs – are more often being underreported compared to healthier foods such as fruit, vegetables, fish etc [41]. This could possibly have led to an underestimation of the UPF intake. However, a previous publication from the VeggiSkills-Sweden study [14] showed that only 17 (7%) of the entire study population underreported their intake in the 24 h-recalls and the percentage did not differ between the dietary groups. Thus, this should not have had any greater impact on our results.

Another limitation is the recruitment method based on convenience sampling, possibly leading to a non-representative sample of the target population. Most likely, this study included a higher proportion of individuals with an interest in diet and health than is true for the youth population in general. This would affect the generalizability of the study results. Finally, for this publication, the only indicators of health investigated were BMI, dietary intake and nutrient status. Thus, no conclusions regarding UPF intake and other health indicators investigated in previous UPF studies, e.g., glucose levels, cardiovascular health and body composition, can be drawn.

A strength of this study was the similar participant characteristics across the dietary groups, such as age, sex and number of participants, making it possible to do valid comparisons between the dietary groups despite not adjusting for possible confounders. Another strength was comprehensive dietary intake data and rigorously conducted classification using both a conservative and an experimental approach. Almost all 244 participants performed four detailed 24-h recalls which together covered all days of the week as well as the whole year, a proper dietary assessment method for the purpose [42]. Both dietary group and the participants’ notes (although not carried out by all participants) were considered during UPF classification of consumed foods instead of merely classifying each unique food item the same.

## Conclusions

This cross-sectional study showed that youth adhering to a vegan diet obtained more energy and nutrients from UPFs than those adhering to lacto-ovo-vegetarian, pescatarian and omnivore diets. Almost all vitamin D and B_12_ were obtained from UPFs among those consuming a vegan diet. Further, the vegans’ intake of total energy, salt and free sugars, and BMI, did not differ from the other dietary groups. Thus, despite the higher UPF consumption among vegans, they did not seem to eat unhealthier than those consuming less strict plant-based or omnivore diets. This study indicates that authorities should be careful discouraging intake of UPFs, as it is defined today, while at the same time promoting a more plant-based diet.

## Supplementary Information

Supplementary Material 1. Supplemental Table 2. Percentage contribution (%) of UPFs to weight of total food and beverage intake, and total energy and nutrient intake in 16–24-year-olds participating in the VeggiSkills-Sweden study using an experimental approach during UPF classification.

Supplementary Material 2. Supplemental Table 1. Absolute and relative mean intake of UPFs (using a conservative approach for UPF classification) as weight of total food and beverage, energy, nutrients, whole grain, sugar and salt in 16-24-year-olds participating in the VeggiSkills-Sweden study.

Supplementary Material 3. Supplemental Table 3. Associations between UPF intake and participant characteristics, and UPF intake and dietary indicators for a healthy/unhealthy food intake in 16-24-year-old vegans, lacto-ovo-vegetarians and pescatarians in the VeggiSkills-Sweden study, using a conservative approach when classifying UPFs.

Supplementary Material 4. Supplemental table 5. Nutrient status by quartiles/tertiles of UPF intake using a conservative approach when classifying UPFs in 16-24-year-olds participating in the VeggiSkills-Sweden study.

Supplementary Material 5. Supplemental Table 4. Associations between UPF intake and participant characteristics, and UPF intake and dietary indicators for a healthy/unhealthy food intake in 16-24-year-old lacto-ovo-vegetarians and pescatarians in the VeggiSkills-Sweden study, using a conservative approach when classifying UPFs.

Supplementary Material 6. Supplemental Table 6. Proportion of 16-24-year-olds at risk of nutrient deficiency according to UPF intake using a conservative approach when classifying UPFs in the VeggiSkills-Sweden study.

Supplementary Material 7. Supplementary text 1. Deviations from the analysis plan.

Supplementary Material 8. Supplemental Figure 1 Haemoglobin levels across the tertiles (T) of ultra-processed food intake (E%) in 16-24-year-old vegans (n=58) participating in the VeggiSkills-Sweden study

Supplementary Material 9. STROBE checklist for reporting observational studies was used and a STROBE statement has been submitted.

## Data Availability

The data generated and analyzed in the current study is not publicly available due to ongoing analysis. However, data can be made available on reasonable request.

## Abbreviations

25-OH-D_3_: 25-hydroxyvitamin D3
BMI: Body mass index
Hb: Haemoglobin
MMA: Methylmalonic acid
MUFA: Monounsaturated fatty acids
NNR: Nordic nutrition recommendations
PAL: Physical activity level
PBMA: Plant-based meat analogue
PUFA: Polyunsaturated fatty acids
SFA: Saturated fatty acids
sTfR: Serum transferrin receptor
UPF: Ultra-processed food
WHO: World Health Organization
