# Supplementary material for: Ultra-processed food intake and its nutritional consequences among youth when consuming vegan, lacto-ovo-vegetarian, pescatarian or omnivore diets: a cross-sectional study
Source: Nutr J. 2026 Aug 4;25:83. doi: 10.1186/s12937-026-01378-8 (PMC13435492; doi:10.1186/s12937-026-01378-8)
Supplement: Supplementary file 1 [file 12937_2026_1378_MOESM1_ESM.pdf]

Supplemental Table 2 Percental contribution (%) of UPFs to weight of total food and beverage intake, and total energy and nutrient intake in 16–24-year-olds participating in the VeggiSkills-Sweden study using an **experimental approach** during UPF classification.

	All participants (n=235)	Vegan (n=60)	Lacto-ovo-vegetarian (n=59)	Pescatarian (n=55)	Omnivore (n=61)	p-value[*]
	Median (IQR)	Median (IQR)	Median (IQR)	Median (IQR)	Median (IQR)	
Weight of food and beverages	27.9 (19.4; 36.8)	30.0 (20.5; 42.6)	26.0 (19.7; 37.1)	27.9 (21.4; 33.0)	26.1 (18.8; 37.3)	0.379
Energy	49.1 (41.5; 58)	52.2 (45.3; 62.9)	47.1 (39.4; 58.6)	47.0 (40.5; 54.7)	48.3 (38.9; 55.8)	0.055
Protein	43.7 (33.8; 57.2)	55.9 (41.7; 63.1)[a]	44.9 (37.1; 57.2)[ab]	39.8 (32.6; 45.4)[b]	36.4 (27.7; 46.9)[c]	**<0.001**
Fat	50.4 (41.0; 61.4)	59.8 (49.6; 67.5)[a]	50.5 (39.4; 60.4)[b]	46.5 (38.6; 56.0)[b]	48.6 (36.8; 56.5)[b]	**<0.001**
SFA	52.4 (39.4; 70.4)	74.8 (61.6; 82.5)[a]	48.9 (36.2; 63.3)[b]	43.0 (35.7; 56.6)[b]	44.8 (38.3; 58.2)[b]	**<0.001**
MUFA	50.5 (39.1; 60.6)	53.4 (43.3; 64.0)	51.4 (37.7; 59.3)	49.4 (37.5; 57.8)	49.6 (36.2; 63.1)	0.239
PUFA	54.7 (40.9; 63.8)	57.8 (45.5; 66.3)	55.2 (43.5; 67.1)	50.1 (36.9; 61.1)	52.7 (39.4; 63.8)	0.169
n-3 fatty acids[†]	50.7 (35.9; 68.0)	61.0 (45.7; 75.8)[a]	56.7 (38.7; 65.3)	42.5 (29.6; 61.1)[b]	47.5 (32.1; 66.3)[b]	**0.002**
Carbohydrates	51.5 (41.1; 60.7)	50.7 (40.1; 57.9)	52.1 (41.5; 61.4)	49.5 (42.7; 60.7)	52.9 (40.3; 61.8)	0.885
Dietary fiber	42.1 (31.3; 54.9)	42.5 (34.8; 53.1)	42.6 (31.8; 58.9)	41.3 (27.9; 55.8)	41.7 (29.3; 52.2)	0.698
Whole grain[‡]	34.4 (13.9; 68.1)	40.0 (17.6; 78.7)	34.2 (16.5; 67.9)	31.7 (9.4; 62.0)	32.7 (11.4; 59.3)	0.345
Free sugars[§]	92.2 (76.2; 97.9)	91.5 (77.2; 97.0)	91.3 (74.3; 98.6)	94.3 (78.8; 98.0)	92.3 (74.7; 97.1)	0.902
Salt	53.4 (41.0; 62.0)	58.4 (47.6; 70.3)[a]	55.5 (41.6; 60.2)	46.4 (36.9; 56.6)[b]	53.8 (37.7; 65.3)	**<0.001**
Vitamin A	25.8 (17.1; 43.1)	34.8 (18.5; 51.8)	25.8 (14.3; 38.6)	22.6 (16.7; 34.2)	25.5 (18.3; 45.3)	0.156
Vitamin D	57.5 (30.4; 91.8)	98.1 (90.4; 100)[a]	63.2 (36.1; 88.0)[b]	35.5 (15.5; 63.3)[c]	44.5 (20.7; 55.9)[cd]	**<0.001**
Vitamin E	53.2 (38.6; 64.4)	60.0 (47.2; 67.4)[a]	54.7 (37.5; 65.4)	47.1 (34.6; 58.9)[b]	47.3 (35.4; 63.7)[b]	**0.004**
Vitamin K	18.5 (8.6; 30.3)	15.9 (8.1; 28.4)	19.0 (9.2; 30.3)	21.1 (7.6; 32.8)	19.9 (8.6; 31.8)	0.754
Thiamine	47.7 (34.2; 59.8)	53.1 (40.6; 60.1)[a]	50.2 (34.8; 62.7)	45.3 (33.3; 59.9)	40.6 (29.3; 52.9)[b]	**0.008**
Riboflavin	52.8 (39.5; 66.1)	65.4 (55.8; 75.4)[a]	50.3 (38.9; 64.8)[b]	48.2 (36.1; 56.0)[b]	45.7 (30.8; 56.8)[b]	**<0.001**
Vitamin C	17.2 (9.0; 32.2)	18.6 (12.3; 36.5)	14.8 (7.2; 25.2)	16.2 (8.8; 38.3)	21.7 (9.2; 42.3)	0.182
Niacin	39.0 (28.1; 50.1)	42.8 (33.9; 55.5)[a]	39.1 (27.2; 49.3)	34.3 (28.8; 47.2)	34.5 (24.5; 46.1)[b]	**0.022**
Vitamin B_6	38.1 (28.0; 51.1)	41.2 (30.4; 53.1)	38.2 (27.9; 52.1)	37.6 (28.5; 53.4)	35.0 (24.7; 48.2)	0.375
Vitamin B_{12}	53.9 (27.4; 87.9)[‖]	100 (96.5; 100)[‖a]	51.8 (31.0; 70.5)[b]	28.0 (16.8; 54.3)[c]	35.7 (24.8; 54.3)[bc]	**<0.001**[‖]
Folate	37.5 (27.7; 49.0)	41.0 (29.7; 49.7)	36.9 (28.4; 53.6)	36.5 (26.7; 48.3)	35.0 (25.4; 47.7)	0.216

Iodine	24.4 (14.2; 38.6)	32.3 (19.0; 46.4)[a]	24.4 (14.0; 37.6)	19.9 (13.3; 26.4)[b]	24.4 (13.2; 39.9)	**0.002**
Phosphorus	40.3 (32.8; 53.6)	49.2 (38.4; 60.9)[a]	45.7 (32.8; 55.7)	37.1 (31.9; 44.7)[b]	35.4 (28.8; 46.0)[b]	**<0.001**
Iron	46.3 (35.6; 58.6)	48.0 (34.2; 59.3)	46.9 (34.1; 60.5)	43.7 (37.1; 55.7)	45.6 (36.9; 58.3)	0.717
Calcium	41.9 (30.6; 54.8)	54.6 (42.8; 66.0)[a]	41.9 (30.7; 54.5)[b]	34.2 (26.2; 50.5)[b]	38.2 (22.6; 48.9)[b]	**<0.001**
Potassium	38.0 (28.5; 47.2)	40.8 (31.3; 49.2)	38.4 (27.8; 51.2)	34.9 (30.0; 43.5)	37.8 (26.0; 44.6)	0.514
Magnesium	38.3 (29.6; 48.6)	40.5 (30.1; 49.5)	39.4 (30.5; 53.3)	37.9 (29.6; 48.2)	37.8 (27.4; 45.8)	0.555
Sodium	52.8 (40.5; 61.0)	56.6 (46.3; 68.2)[a]	53.6 (41.0; 60.1)	45.0 (36.9; 56.1)	52.6 (37.3; 64.8)[b]	**0.001**
Selenium	32.2 (22.3; 48.2)	40.1 (29.4; 52.7)[a]	36.5 (25.8; 52.5)[ab]	25.6 (18.4; 34.0)[c]	30.3 (20.0; 46.1)[bc]	**<0.001**
Zinc	38.4 (30.3; 49.1)	45.8 (33.7; 52.7)[a]	42.5 (30.3; 47.8)	35.1 (29.9; 48.2)	35.4 (26.2; 46.0)[b]	**0.010**

UPF Ultra-processed food

Significant p-values (p<0.05) are in bold.

[*]Differences between the dietary groups were tested using Kruskal Wallis test with Bonferroni post-hoc test to adjust for multiple comparisons. Superscript letters indicate significant differences between groups. Groups sharing the same letter are not significantly different from each other, groups with different letters differ significantly. No superscript letters = no significant differences between any groups.

[†]The sum of alfa-linolenic acid, linoleic acid, eicosapentaenoic acid, docohexaenoic acid, and docosapentaenoic acid

[‡]Missing data due to no total intake of whole grain (total n=5, vegan n=2, lacto-ovo-vegetarian n=2, pescetarian n=1)

[§]Missing data due to no total intake of free sugars (omnivore n=1)

[‖]One participant (vegan) removed from analyses due to incorrect inclusion of vitamin B_{12} supplement in the dataset.
