# Supplementary material for: Ultra-processed food intake and its nutritional consequences among youth when consuming vegan, lacto-ovo-vegetarian, pescatarian or omnivore diets: a cross-sectional study
Source: Nutr J. 2026 Aug 4;25:83. doi: 10.1186/s12937-026-01378-8 (PMC13435492; doi:10.1186/s12937-026-01378-8)
Supplement: Supplementary file 2 [file 12937_2026_1378_MOESM2_ESM.pdf]

Supplemental Table 1 Absolute and relative mean intake of UPFs (using a conservative approach for UPF classification) as weight of total food and beverage, energy, nutrients, whole grain, sugar and salt in 16-24-year-olds participating in the VeggiSkills-Sweden study.

	**All participants (n=235)**	**Vegan (n=60)**	**Lacto-ovo-vegetarian (n=59)**	**Pescatarian (n=55)**	**Omnivore (n=61)**
	Median (IQR)	Median (IQR)	Median (IQR)	Median (IQR)	Median (IQR)
Weight of food and beverages, g	718 (497; 975)	866 (627; 1129)	718 (464; 856)	668 (453; 874)	656 (437; 960)
Energy, kcal	819 (605; 1091)	1027 (765; 1242)	785 (576; 1103)	776 (591; 876)	790 (595; 998)
Energy, MJ	3.4 (2.5; 4.6)	4.3 (3.2; 5.2)	3.3 (2.4; 4.6)	3.2 (2.5; 3.7)	3.3 (2.5; 4.2)
Protein, g	26 (17; 36)	34 (19; 42)	27 (16; 38)	21 (17; 30)	24 (15; 35)
Protein, E%	5 (3; 7)	6 (4; 7)	5 (3; 7)	4 (3; 6)	4 (3; 6)
Fat, g	36 (26; 51)	46 (36; 61)	32 (22; 53)	35 (25; 44)	36 (24; 52)
Fat, E%	15 (11; 19)	18 (14; 23)	14 (10; 18)	14 (11; 17)	14 (10; 17)
SFA, g	11 (8; 16)	14 (9; 19)	10 (7; 13)	11 (8; 14)	12 (8; 16)
SFA, E%	5 (3; 6)	5 (4; 7)	4 (3; 5)	5 (3; 6)	5 (3; 6)
MUFA, g	15 (10; 20)	17 (13; 22)	13 (9; 21)	14 (10; 18)	16 (10; 20)
MUFA, E%	6 (4; 8)	7 (6; 9)	5 (4; 8)	6 (4; 8)	6 (4; 7)
PUFA, g	7 (4; 11)	11 (7; 13)	7 (4; 10)	6 (4; 8)	5 (4; 8)
PUFA, E%	3 (2; 4)	4 (3; 6)	3 (2; 4)	2 (2; 3)	2 (1; 3)
n-3 fatty acids, g	1 (0.5; 1.5)	1.4 (0.8; 2)	0.9 (0.6; 1.6)	0.9 (0.5; 1.3)	0.8 (0.3; 1.2)
n-3 fatty acids, E%	0.4 (0.2; 0.6)	0.5 (0.4; 0.8)	0.4 (0.2; 0.7)	0.4 (0.2; 0.5)	0.3 (0.1; 0.5)
Carbohydrates, g	87 (60; 118)	107 (71; 139)	81 (48; 110)	76 (60; 101)	87 (53; 118)
Carbohydrates, E%	16 (12; 21)	20 (14; 25)	16 (11; 21)	16 (12; 20)	15 (11; 19)
Dietary fiber, g	8 (5; 12)	12 (9; 15)	8 (6; 12)	7 (5; 9)	6 (4; 9)
Dietary fiber, g/MJ	0.9 (0.6; 1.2)	1.3 (1; 1.7)	0.9 (0.6; 1.2)	0.7 (0.6; 1)	0.6 (0.4; 1)
Whole grain, g	6 (0; 16)	10 (1; 16)	5 (1; 12)	4 (0; 16)	5 (0; 16)
Free sugars, g	13 (5; 23)	13 (4; 21)	12 (5; 23)	16 (9; 23)	13 (4; 26)
Free sugars, E%	2 (1; 4)	2 (1; 4)	2 (1; 4)	3 (2; 5)	2 (1; 4)
Salt, g	3.3 (2.4; 4.6)	4 (3.3; 6.3)	3.2 (2.4; 4.6)	2.7 (2; 3.7)	3.3 (2.5; 4.7)
Alcohol, g	0 (0; 0)	0 (0; 0)	0 (0; 1.1)	0 (0; 0)	0 (0; 0.01)
Vitamin A, RE	119 (74; 199)	131 (60; 211)	113 (66; 174)	119 (82; 200)	118 (80; 203)

Vitamin A, RE/MJ	14 (8; 21)	13 (7; 24)	13 (8; 20)	14 (11; 20)	14 (8; 20)
Vitamin D, µg	2 (1; 4)	3 (2; 5)	2 (1; 3)	2 (1; 3)	2 (1; 4)
Vitamin D, µg/MJ	0.2 (0.1; 0.4)	0.3 (0.2; 0.6)	0.2 (0.1; 0.4)	0.2 (0.1; 0.3)	0.2 (0.1; 0.4)
Vitamin E, mg	7 (5; 11)	10 (7; 15)	7 (5; 11)	7 (4; 9)	6 (4; 9)
Vitamin E, mg/MJ	0.8 (0.6; 1.1)	1.1 (0.8; 1.5)	0.9 (0.5; 1.2)	0.7 (0.5; 1)	0.6 (0.4; 0.8)
Vitamin K, µg	11 (6; 17)	11 (7; 18)	13 (6; 19)	10 (6; 15)	10 (4; 17)
Vitamin K, µg/MJ	1.2 (0.7; 1.9)	1.2 (0.8; 1.8)	1.3 (0.7; 2.1)	1.2 (0.7; 2)	1.1 (0.5; 1.9)
Thiamine, mg	0.4 (0.3; 0.7)	0.7 (0.4; 0.8)	0.4 (0.3; 0.7)	0.3 (0.2; 0.5)	0.4 (0.2; 0.6)
Thiamine, mg/MJ	0.04 (0.03; 0.07)	0.07 (0.05; 0.1)	0.04 (0.03; 0.06)	0.04 (0.03; 0.06)	0.04 (0.03; 0.05)
Riboflavin, mg	0.6 (0.4; 0.8)	0.7 (0.4; 0.9)	0.5 (0.4; 0.8)	0.5 (0.3; 0.7)	0.6 (0.3; 0.8)
Riboflavin, mg/MJ	0.06 (0.04; 0.09)	0.07 (0.05; 0.1)	0.06 (0.04; 0.09)	0.06 (0.04; 0.08)	0.06 (0.03; 0.09)
Vitamin C, mg	13 (6; 24)	18 (8; 33)	10 (4; 17)	11 (7; 23)	15 (5; 32)
Vitamin C, mg/MJ	1.4 (0.7; 2.5)	1.8 (0.8; 3.3)	1.1 (0.5; 1.9)	1.1 (0.8; 2.5)	1.6 (0.5; 3.4)
Niacin, NE	7.5 (5; 12.3)	8.1 (5.8; 12.9)	6.8 (3.7; 11)	6.7 (4.5; 9.5)	8.5 (5.5; 14.5)
Niacin, NE/MJ	0.8 (0.5; 1.2)	0.9 (0.7; 1.2)	0.7 (0.4; 1.1)	0.8 (0.5; 1.1)	0.9 (0.6; 1.6)
Vitamin B_6, mg	0.5 (0.3; 0.8)	0.6 (0.4; 1)	0.5 (0.3; 0.7)	0.4 (0.3; 0.7)	0.5 (0.3; 0.8)
Vitamin B_6, mg/MJ	0.05 (0.03; 0.08)	0.06 (0.05; 0.1)	0.04 (0.03; 0.07)	0.05 (0.03; 0.08)	0.05 (0.03; 0.09)
Vitamin B_{12}, µg	1 (0.5; 1.7)	1.1 (0.5; 1.8)	0.9 (0.5; 1.3)	0.8 (0.4; 1.4)	1.4 (0.6; 1.8)
Vitamin B_{12}, µg/MJ	0.11 (0.06; 0.19)	0.12 (0.06; 0.21)	0.09 (0.05; 0.17)	0.09 (0.04; 0.17)	0.11 (0.07; 0.21)
Folate, µg	104 (65; 149)	143 (99; 198)	118 (67; 164)	83 (62; 112)	84 (48; 111)
Folate, µg/MJ	11 (7; 16)	15 (11; 20)	12 (8; 17)	9 (6; 14)	8 (5; 12)
Iodine, µg	35 (20; 51)	37 (23; 64)	34 (17; 49)	36 (20; 48)	29 (17; 50)
Iodine, µg/MJ	4 (2; 6)	4 (2; 7)	3 (2; 7)	4 (2; 6)	3 (2; 5)
Phosphorus, mg	441 (312; 594)	496 (387; 644)	445 (327; 594)	387 (302; 514)	407 (269; 565)
Phosphorus, mg/MJ	47 (34; 63)	56 (38; 68)	49 (31; 63)	45 (33; 62)	43 (30; 52)
Iron, mg	4 (3; 5)	5 (4; 7)	4 (2; 6)	3 (2; 4)	3 (2; 5)
Iron, mg/MJ	0.4 (0.3; 0.6)	0.6 (0.4; 0.7)	0.4 (0.3; 0.6)	0.3 (0.2; 0.5)	0.4 (0.3; 0.5)
Calcium, mg	303 (186; 437)	337 (214; 448)	339 (232; 486)	284 (196; 457)	261 (131; 378)
Calcium, mg/MJ	32 (21; 46)	34 (23; 54)	38 (25; 51)	31 (22; 50)	25 (15; 39)
Potassium, mg	825 (630; 1153)	1050 (753; 1294)	796 (559; 1233)	772 (605; 991)	825 (606; 1084)
Potassium, mg/MJ	93 (69; 121)	115 (80; 132)	89 (65; 135)	85 (72; 108)	83 (64; 110)
Magnesium, mg	107 (75; 161)	146 (114; 190)	116 (77; 165)	94 (75; 132)	91 (57; 126)
Magnesium, mg/MJ	12 (8; 17)	16 (12; 20)	12 (9; 18)	11 (8; 15)	9 (7; 13)

Sodium, mg	1337 (1002; 1846)	1632 (1250; 2433)	1313 (1027; 1830)	1081 (835; 1532)	1294 (1038; 1890)
Sodium, mg/MJ	149 (109; 199)	173 (145; 236)	145 (107; 204)	125 (90; 172)	146 (108; 190)
Selenium, µg	8 (5; 12)	9 (5; 12)	7 (5; 11)	7 (4; 9)	10 (5; 13)
Selenium, µg/MJ	0.8 (0.5; 1.2)	0.9 (0.6; 1.4)	0.8 (0.5; 1.2)	0.6 (0.4; 1)	1 (0.6; 1.4)
Zinc, mg	2.7 (1.8; 3.7)	3 (2.2; 3.7)	2.5 (1.4; 4)	2.4 (1.8; 3.3)	3 (1.7; 3.9)
Zinc, mg/MJ	0.28 (0.2; 0.4)	0.31 (0.25; 0.41)	0.28 (0.15; 0.42)	0.24 (0.17; 0.38)	0.32 (0.21; 0.39)

E% Energy percent, *MJ* Megajoule, *MUFA* Monounsaturated fatty acids, *PUFA* Polyunsaturated fatty acids, *SFA* Saturated Fatty acids, *UPF* Ultra-processed food

For descriptive purposes only; no statistical tests of differences between dietary groups were performed.
