# Supplementary material for: Ultra-processed food intake and its nutritional consequences among youth when consuming vegan, lacto-ovo-vegetarian, pescatarian or omnivore diets: a cross-sectional study
Source: Nutr J. 2026 Aug 4;25:83. doi: 10.1186/s12937-026-01378-8 (PMC13435492; doi:10.1186/s12937-026-01378-8)
Supplement: Supplementary file 4 [file 12937_2026_1378_MOESM4_ESM.pdf]

Supplemental table 5 Nutrient status by quartiles/tertiles of UPF intake using a conservative approach when classifying UPFs in 16-24-year-olds participating in the VeggiSkills-Sweden study.

	E% UPF intake (range)				
	Median (IQR)				p-value[*]
**All participants, n=235[†]**	**Q1 (2.6-29.6)**	**Q2 (29.7-37.3)**	**Q3 (37.4-46.6)**	**Q4 (46.7-81.9)**	
Hb, g/L	138 (132; 145)	139 (133; 150)	141 (133; 150)	140 (128; 146)	0.598
sTfR, mg/L	0.84 (0.7; 1.06)	0.84 (0.73; 1.00)	0.84 (0.69; 0.97)	0.86 (0.72; 0.96)	0.908
tHcy, µmol/L	3.8 (2.9; 5.0)	4.4 (3.3; 5.5)	3.9 (2.9; 5.6)	3.6 (2.6; 5.1)	0.249
MMA, µmol/L	0.10 (0.08; 0.18)	0.11 (0.07; 0.17)	0.10 (0.07; 0.18)	0.11 (0.07; 0.14)	0.658
25-OH-D3, nmol/L	45 (35; 55)	46 (39; 55)	40 (32; 55)	46 (36; 53)	0.398
**Vegan, n=60[‡]**	**T1 (11.8-40.6)**	**T2 (41.8-48.4)**	**T3 (48.9-81.9)**		
Hb, g/L	147 (137; 156)[a]	135 (128; 144)[b]	141 (129; 146)		**0.025**
sTfR, mg/L	0.92 (0.79; 1.16)	0.88 (0.72; 0.96)	0.86 (0.75; 0.97)		0.484
tHcy, µmol/L	4.4 (2.9; 5.9)	3.5 (2.6; 5.1)	3.4 (2.5; 4.4)		0.358
MMA, µmol/L	0.13 (0.07; 0.18)	0.13 (0.07; 0.2)	0.13 (0.11; 0.15)		0.973
25-OH-D3, nmol/L	43 (30; 55)	36 (27; 48)	37 (26; 52)		0.446
**Lacto-ovo-vegetarian, n=59[§]**	**T1 (12.2-28.4)**	**T2 (28.5-41.3)**	**T3 (41.5-71.2)**		
Hb, g/L	135 (128; 144)	140 (136; 147)	141 (125; 153)		0.374
sTfR, mg/L	0.93 (0.79; 1.32)	0.8 (0.73; 0.95)	0.76 (0.72; 0.92)		0.109
tHcy, µmol/L	4 (2.9; 5)	4.6 (3.3; 5.5)	4.9 (3.3; 7.1)		0.113
MMA, µmol/L	0.09 (0.06; 0.13)	0.17 (0.11; 0.24)	0.09 (0.07; 0.26)		0.077
25-OH-D3, nmol/L	43 (32; 51)	40 (33; 53)	48 (36; 53)		0.458
**Pescatarian, n=55[‖]**	**T1 (17.4-32.2)**	**T2 (32.5-40.4)**	**T3 (40.9-63.7)**		
Hb, g/L	138 (131; 141)	140 (127; 150)	138 (128; 148)		0.888
sTfR, mg/L	0.88 (0.79; 1.23)	0.91 (0.73; 1.08)	0.74 (0.58; 0.89)		0.086
tHcy, µmol/L	4.5 (3.8; 5.5)	4.6 (3; 5.7)	4.4 (3.2; 5.8)		0.922
MMA, µmol/L	0.11 (0.08; 0.17)	0.12 (0.07; 0.19)	0.1 (0.06; 0.24)		0.961
25-OH-D3, nmol/L	54 (39; 66)	50 (44; 59)	53 (43; 57)		0.877

Omnivore. n=61[¶]	T1 (2.6-29.8)	T2 (30.6-41.0)	T3 (41.7-72.5)		
Hb, g/L	138 (135; 145)	142 (133; 151)	141 (130; 150)		0.677
sTfR, mg/L	0.73 (0.59; 0.83)	0.85 (0.72; 0.97)	0.87 (0.7; 1.08)		0.063
tHcy, µmol/L	3.4 (2.7; 4.1)	3.6 (2.7; 4.7)	3.3 (2.6; 4.4)		0.669
MMA, µmol/L	0.1 (0.08; 0.22)	0.1 (0.07; 0.17)	0.09 (0.06; 0.13)		0.440
25-OH-D3, nmol/L	43 (34; 53)	44 (38; 53)	45 (31; 61)		0.974

25-OH-D3 25-hydroxyvitamin D3, *Hb* Haemoglobin, *MMA* Methylmalonic acid, *n/a* non-applicable, *Q* Quartile, *sTfR* Serum transferrin receptor, *T* Tertile, *tHcy* Homocysteine, *UPF* Ultra-processed food

Significant p-values ($p<0.05$) are in bold. Note that many participants, especially vegans, used supplements.
[*]Differences between the quartiles/tertiles were tested using Kruskal Wallis test with Bonferroni post-hoc test to adjust for multiple comparisons. Superscript letters indicate significant differences between groups. Groups sharing the same letter are not significantly different from each other, groups with different letters differ significantly. No superscript letters = no significant differences between any groups.

[†]Missing data: Hb, n=5; sTfR, n=3; tHcy, n=5; MMA, n=45; Vitamin D, n=16
[‡]Missing data: Hb, n=2; tHcy, n=3; MMA, n=12; Vitamin D, n=10
[§]Missing data: sTfR, n=1; tHcy, n=1; MMA, n=11; Vitamin D, n=4
[‖]Missing data: Hb, n=2; sTfR, n=1; tHcy, n=1; MMA, n=11
[¶]Missing data: Hb, n=1; sTfR, n=1; MMA, n=11; Vitamin D, n=2
