# Supplementary material for: Ultra-processed food intake and its nutritional consequences among youth when consuming vegan, lacto-ovo-vegetarian, pescatarian or omnivore diets: a cross-sectional study
Source: Nutr J. 2026 Aug 4;25:83. doi: 10.1186/s12937-026-01378-8 (PMC13435492; doi:10.1186/s12937-026-01378-8)
Supplement: Supplementary file 5 [file 12937_2026_1378_MOESM5_ESM.pdf]

**Supplemental Table 4** Associations between UPF intake and participant characteristics, and UPF intake and dietary indicators for a healthy/unhealthy food intake in 16-24-year-old **lacto-ovo-vegetarians and pescatarians** in the VeggiSkills-Sweden study, using a conservative approach when classifying UPFs.

| | E% UPF intake (range) | | | p-value |
|---|---|---|---|---|
| **Lacto-ovo-vegetarian, n=59** | **T1 (12.2-28.4)** | **T2 (28.5-41.3)** | **T3 (41.5-71.2)** | |
| Sex | | | | 0.572* |
| Female | 17 (90) | 17 (85) | 15 (75) | |
| Male | 2 (11) | 3 (15) | 5 (25) | |
| Age, years | 21 (20; 22) | 23 (20; 24) | 22 (21; 23) | 0.127† |
| Adherence to dietary regimen | | | | 0.197‡ |
| <5 years | 9 (47) | 13 (65) | 15 (75) | |
| ≥5 years | 10 (53) | 7 (35) | 5 (25) | |
| General nutrition knowledge score | 52 (45; 58) | 49 (47; 62) | 47 (42; 54) | 0.238† |
| General nutrition knowledge level | | | | 0.558* |
| Low level | 3 (16) | 2 (10) | 5 (25) | |
| Moderate level | 10 (53) | 11 (55) | 12 (60) | |
| High level | 6 (32) | 7 (35) | 3 (15) | |
| Body mass index, kg/m$^2$ | 20.5 (19.9; 23.5) | 21.5 (20.5; 22.8) | 22.1 (20.6; 23.8) | 0.573† |
| Body mass index category | | | | 0.393* |
| Underweight (BMI <18.5) | 0 (0) | 2 (10) | 3 (15) | |
| Normal weight (BMI 18.5-24.9) | 16 (84) | 17 (85) | 14 (70) | |
| Pre-obesity/Obesity (BMI ≥25) | 3 (16) | 1 (5) | 3 (15) | |
| Physical Activity Level | | | | 0.984* |
| 1.4 or 1.55 | 5 (26) | 6 (30) | 4 (20) | |

| | | | | |
|---|---|---|---|---|
| 1.7 | 10 (53) | 10 (50) | 11 (55) | |
| 1.8 or 2.0 | 4 (21) | 4 (20) | 5 (25) | |
| Energy intake, kcal | 2033 (1618; 2549) | 2090 (1896; 2711) | 2087 (1865; 2415) | 0.683† |
| Whole grain intake, g | 48 (27; 62) | 42 (19; 65) | 29 (4; 47) | 0.073† |
| Dietary fiber intake, g | 27 (22; 36) | 28 (25; 31) | 27 (22; 34) | 0.795† |
| Monounsaturated fatty acids intake, E% | 14 (13; 17) | 15 (13; 17) | 16 (13;18) | 0.749† |
| Polyunsaturated fatty acids intake, E% | 6 (6; 7) | 7 (6; 9) | 8 (6; 9) | 0.249† |
| Saturated fatty acids intake, E% | 13 (11; 17) | 11 (9; 14) | 11 (8; 13) | 0.108† |
| Free sugars intake, E% | 4 (2; 5) | 3 (2; 5) | 4 (3; 6) | 0.429† |
| Salt intake, g | 7 (6; 10) | 10 (7; 12) | 9 (7; 11) | 0.224† |
| | | | | |
| **Pescatarian, n=55** | **T1 (17.4-32.2)** | **T2 (32.5-40.4)** | **T3 (40.9-63.7)** | |
| Sex | | | | 1.000* |
| Female | 16 (89) | 16 (84) | 15 (83) | |
| Male | 2 (11) | 3 (16) | 3 (17) | |
| Age, years | 21 (19; 23) | 21 (20; 23) | 21 (20; 22) | 0.860† |
| Adherence to dietary regimen | | | | 0.510‡ |
| <5 years | 11 (61) | 8 (42) | 9 (50) | |
| ≥5 years | 7 (39) | 11 (58) | 9 (50) | |
| General nutrition knowledge score | 53 (49; 61)[a] | 56 (47; 59) | 48 (36; 52)[b] | **0.020**† |
| General nutrition knowledge level | | | | 0.139* |
| Low level | 1 (6) | 2 (11) | 6 (33) | |
| Moderate level | 10 (56) | 12 (63) | 10 (56) | |
| High level | 7 (39) | 5 (26) | 2 (11) | |

| | | | | |
|---|---|---|---|---|
| Body mass index, $kg/m^2$ | 20.6 (19.7; 22.9) | 21.8 (20.8; 24.1) | 22.4 (20.8; 24.0) | 0.100[†] |
| Body mass index category | | | | 0.549[*] |
| Underweight (BMI <18.5) | 3 (17) | 1 (5) | 0 (0) | |
| Normal weight (BMI 18.5-24.9) | 12 (67) | 14 (74) | 15 (83) | |
| Pre-obesity/Obesity (BMI ≥25) | 2 (17) | 4 (21) | 3 (17) | |
| Physical Activity Level | | | | 0.369[*] |
| 1.4 or 1.55 | 4 (22) | 3 (16) | 8 (44) | |
| 1.7 | 7 (39) | 10 (53) | 6 (33) | |
| 1.8 or 2.0 | 7 (39) | 6 (32) | 4 (22) | |
| Energy intake, kcal | 2266 (1924; 2375) | 2229 (1813; 2419) | 2010 (1790; 2238) | 0.645[†] |
| Whole grain intake, g | 56 (31; 100)[a] | 35 (9; 59) | 20 (9; 50)[b] | **0.025**[†] |
| Dietary fiber intake, g | 30 (22; 36)[a] | 24 (20; 30) | 21 (18; 25)[b] | **0.034**[†] |
| Monounsaturated fatty acids intake, E% | 15 (13; 16) | 15 (14; 17) | 15 (14; 18) | 0.463[†] |
| Polyunsaturated fatty acids intake, E% | 7 (5; 8) | 7 (6; 7) | 7 (5; 8) | 0.884[†] |
| Saturated fatty acids intake, E% | 13 (10; 14) | 12 (10; 16) | 13 (10; 17) | 0.701[†] |
| Free sugars intake, E% | 4 (2; 5) | 4 (3; 6) | 6 (3; 7) | 0.059[†] |
| Salt intake, g | 9 (6; 9) | 9 (6; 10) | 8 (7; 10) | 0.993[†] |

*E%* Energy percent, *T* Tertile, *UPF* Ultra-processed food

Values are median (IQR) for continuous data and no. (%) for categorical data. Significant p-values ($p<0.05$) are in bold.

[*]Differences between the quartiles were tested using Fisher-Freeman-Halton Exact test.

[†]Differences between the quartiles were tested using Kruskal Wallis test with Bonferroni post-hoc test to adjust for multiple comparisons. Superscript letters indicate significant differences between groups. Groups sharing the same letter are not significantly different from each other, groups with different letters differ significantly. No superscript letters = no significant differences between

any groups.
‡Differences between the tertiles were tested using $\chi^2$ test
