# Supplementary material for: Ultra-processed food intake and its nutritional consequences among youth when consuming vegan, lacto-ovo-vegetarian, pescatarian or omnivore diets: a cross-sectional study
Source: Nutr J. 2026 Aug 4;25:83. doi: 10.1186/s12937-026-01378-8 (PMC13435492; doi:10.1186/s12937-026-01378-8)
Supplement: Supplementary file 6 [file 12937_2026_1378_MOESM6_ESM.pdf]

Supplemental Table 6 Proportion of 16-24-year-olds at risk of nutrient deficiency according to UPF intake using a conservative approach when classifying UPFs in the VeggiSkills-Sweden study.

	E% UPF intake (range)				
	No. (%)				p-value
**All participants, n=235***	**Q1 (2.6-29.6)**	**Q2 (29.7-37.3)**	**Q3 (37.4-46.6)**	**Q4 (46.7-81.9)**	
Hb, Risk of anaemia (F: <120 g/L, M: <130 g/L)	0	0	0	0	n/a
sTfR, Elevated status (>3.09 mg/L)	0	0	0	0	n/a
tHcy, Elevated status (>15 µmol/L)	1 (2)	1 (2)	0 (0)	0 (0)	0.617†
MMA, Depletion (>0.27–0.37 µmol/L)	2 (3)	2 (3)	5 (9)	1 (2)	0.363†
MMA, Risk of deficiency (>0.37 µmol/L)	5 (9)	1 (2)	1 (2)	3 (5)	
25-OH-D3, suboptimal status (30-49 nmol/L)	25 (43)	27 (46)	27 (46)	25 (42)	0.839‡
25-OH-D3, Risk of deficiency (<30 nmol/L)	6 (10)	6 (10)	10 (17)	6 (10)	
**Vegan, n=60§**	**T1 (11.8-40.6)**	**T2 (41.8-48.4)**	**T3 (48.9-81.9)**		
Hb, Risk of anaemia (F: <120 g/L, M: <130 g/L)	0 (0)	0 (0)	0 (0)		n/a
sTfR, Elevated status (>3.09 mg/L)	0 (0)	0 (0)	0 (0)		n/a
tHcy, Elevated status (>15 µmol/L)	1 (5)	0 (0)	(0)		1.000†

MMA, Depletion (>0.27–0.37 µmol/L)	2 (10)	1 (5)	0 (0)		0.764†
MMA, Risk of deficiency (>0.37 µmol/L)	0 (0)	0 (0)	1 (5)		
25-OH-D3, suboptimal status (30-49 nmol/L)	5 (25)	11 (55)	5 (25)		0.283†
25-OH-D3, Risk of deficiency (<30 nmol/L)	4 (20)	5 (25)	5 (25)		
**Lacto-ovo-veg, n=50‖**	**T1 (12.2-28.4)**	**T2 (28.5-41.3)**	**T3 (41.5-71.2)**		
Hb, Risk of anaemia (F: <120 g/L, M: <130 g/L)	0 (0)	0 (0)	0 (0)		n/a
sTfR, Elevated status (>3.09 mg/L)	0 (0)	0 (0)	0 (0)		n/a
tHcy, Elevated status (>15 µmol/L)	1 (5)	0 (0)	0 (0)		0.655†
MMA, Depletion (>0.27–0.37 µmol/L)	0 (0)	1 (5)	2 (10)		0.713†
MMA, Risk of deficiency (>0.37 µmol/L)	1 (5)	2 (10)	2 (10)		
25-OH-D3, suboptimal status (30-49 nmol/L)	10 (53)	11 (55)	10 (50)		0.231†
25-OH-D3, Risk of deficiency (<30 nmol/L)	3 (16)	3 (15)	0 (0)		
**Pescatarian; n=55¶**	**T1 (17.4-32.2)**	**T2 (32.5-40.4)**	**T3 (40.9-63.7)**		
Hb, Risk of anaemia (F: <120 g/L, M: <130 g/L)	0 (0)	0 (0)	0 (0)		n/a

sTfR, Elevated status (>3.09 mg/L)	0 (0)	0 (0)	0 (0)		n/a
tHcy, Elevated status (>15 µmol/L)	0 (0)	0 (0)	0 (0)		n/a
MMA, Depletion (>0.27–0.37 µmol/L)	0 (0)	0 (0)	2 (11)		0.542†
MMA, Risk of deficiency (>0.37 µmol/L)	1 (6)	0 (0)	1 (6)		
25-OH-D3, suboptimal status (30-49 nmol/L)	4 (22)	9 (47)	7 (39)		0.279†
25-OH-D3, Risk of deficiency (<30 nmol/L)	3 (17)	0 (0)	1 (6)		
**Omnivore, n=61#**	**T1 (2.6-29.8)**	**T2 (30.6-41.0)**	**T3 (41.7-72.5)**		
Hb, Risk of anaemia (F: <120 g/L, M: <130 g/L)	0 (0)	0 (0)	0 (0)		n/a
sTfR, Elevated status (>3.09 mg/L)	0 (0)	0 (0)	0 (0)		n/a
tHcy, Elevated status (>15 µmol/L)	0 (0)	0 (0)	0 (0)		n/a
MMA, Depletion (>0.27–0.37 µmol/L)	1 (5)	1 (5)	0 (0)		0.185†
MMA, Risk of deficiency (>0.37 µmol/L)	2 (10)	0 (0)	0 (0)		
25-OH-D3, suboptimal status (30-49 nmol/L)	11 (55)	10 (48)	11 (55)		1.000†
25-OH-D3, Risk of deficiency (<30 nmol/L)	1 (5)	2 (10)	1 (5)		

25-OH-D3 25-hydroxyvitamin D3, *F* Female, *Hb* Haemoglobin, *M* Male, *MMA* Methylmalonic acid, *n/a* non-applicable, *sTfR* Serum transferrin receptor, *tHcy* Homocysteine, *UPF* Ultra-processed food

[*]Missing data: Hb, n=5; sTfR, n=3; tHcy, n=5; MMA, n=45; Vitamin D, n=16
[†]Differences between the quartiles/tertiles were tested using Fisher-Freeman-Halton Exact test
[‡]Differences between the quartiles/tertiles were tested using χ^2 test
[§]Missing data: Hb, n=2; tHcy, n=3; MMA, n=12; Vitamin D, n=10
[‖]Missing data: sTfR, n=1; tHcy, n=1; MMA, n=11; Vitamin D, n=4
[¶]Missing data: Hb, n=2; sTfR, n=1; tHcy, n=1; MMA, n=11
[#]Missing data: Hb, n=1; sTfR, n=1; MMA, n=11; Vitamin D, n=2
