# Supplementary material for: Ultra-processed food intake and its nutritional consequences among youth when consuming vegan, lacto-ovo-vegetarian, pescatarian or omnivore diets: a cross-sectional study
Source: Nutr J. 2026 Aug 4;25:83. doi: 10.1186/s12937-026-01378-8 (PMC13435492; doi:10.1186/s12937-026-01378-8)
Supplement: Supplementary file 7 [file 12937_2026_1378_MOESM7_ESM.pdf]

Supplementary Text 1 Deviations from the analysis plan

When classifying tea and tomato paste, the analysis plan (Sällström and Bärebring, unpublished) was mistakenly disregarded. Due to the most popular teas containing aromas (a UPF-marker) and tomato paste containing concentrate they were here classified as UPFs which could be considered incorrect. Martinez-Steele et al classify tea as “Unprocessed and minimally processed foods” and no other concentrate except from fruit juice concentrate is identified as UPF-markers [21]. To some degree, these possibly incorrect classifications may have overestimated UPF intake. Since tea is energy free and nutrient-poor, the overestimation only affects the interpretation of the results of the UPF contribution to total weight of food and beverages analyses. In addition, consumption of tea and coffee intake did not differ between the dietary groups (data not shown). Tomato paste is not consumed in a very large amount and therefore does not contribute much to energy and nutrient intake. Since tomato paste was classified as UPF, this was also considered a UPF-ingredient in composite dishes. In industrially manufactured dishes containing tomato paste, there are often other UPF-markers as well. Also, there is no reason to believe that the intake of tomato paste and dishes containing tomato paste would differ between the dietary groups. Therefore, we do not believe that the incorrect classifications of tea and tomato paste had any significant effects on the outcomes.
